# Spatial organisation within the earliest evidence of post-built structures in Britain

**DOI:** 10.1371/journal.pone.0306908

**Published:** 2024-07-15

**Authors:** Jessica Bates, Nicky Milner, Chantal Conneller, Aimée Little

**Affiliations:** 1 Department of Archaeology, University of York, York, United Kingdom; 2 School of History, Classics and Archaeology, Newcastle University, Newcastle upon Tyne, United Kingdom; 3 Department of Archaeology, Centre for Artefacts and Materials Analysis, University of York, York, United Kingdom; Tel Aviv university, ISRAEL

## Abstract

This paper explores tool-using activities undertaken in and around the earliest known evidence of post-built structures in Britain. Microwear results associated with at least three structures identified at the Early Mesolithic site of Star Carr, North Yorkshire, are examined as a means of identifying activity zones associated with the diverse stone tools used to process a variety of materials (e.g. wood, bone, antler, plant, hide, meat, fish). With 341 lithic artefacts analysed, this research represents the first microwear study focused on the post-built structures at Star Carr. A combination of spatial and microwear data has provided different scales of interpretation: from individual tool use to patterns of activity across the three structures. Different types of tool use observed have aided interpretations of possible activity areas where objects were produced and materials were processed. Zones of activity within one of the structures suggest that the working of some materials was more spatially restricted than others; even where there are high densities of flint deposition, spatial patterns in tool-using activity were observed. From this, it is interpreted that social norms and behaviours influenced the spatial organisation of different spaces. Our results demonstrate the importance of combining microwear analysis with GIS to explore function and variability in the use of Mesolithic structures—providing new insights into their role as social spaces.

## Introduction

Mesolithic structures can provide critical insights into the daily activities of the individuals who built and inhabited them, depending on the preservation of associated archaeological features and finds. Prior to the application of microwear analysis, archaeologists interpreted Mesolithic settlements through tool types, site context and (where present) faunal remains [1–6]. Tool typologies played an integral role in assigning function to settlements, with certain types associated with particular activities; for example, scrapers were seen as indicative of hide working [7–11]. These were often based on simplistic comparisons between archaeological and ethnographic tools [12,13]. Frequencies of tool types present were then used to infer activities undertaken [14,15]. In doing so, a site could be assigned a ‘type’; for example, butchery, wood working and hunting activities would lead to a categorisation of a home base [15–20]. In the absence of microwear data, more recent studies of Mesolithic settlements continue to implement this approach of inferring activity areas from tool types and assigning site function on this basis [18,21–25]. This has led to broad categorisations of Mesolithic structures, such as home base or hunting camp, which lack an understanding of the variation in the range of tasks that may have been undertaken within these spaces.

Through the application of microwear analysis, the range of activities undertaken in and around structures can be better assessed. Flint microwear can provide insights into the nature of site use and intra-site organisation [e.g. 26–29], as well as inter-site patterns concerning settlement systems and hunter-gatherer movements across regions [e.g. 30,31]. When plotted spatially, microwear analysis can also shed light on patterning in tool-using behaviours, indicating if particular tasks were undertaken in specific areas [32–34]. If applied alongside other complementary methods, such as refitting, technological assessment, analysis of faunal remains and geochemistry of associated soils, an even more holistic understanding of different activity areas can be gained [35].

Owing to the time intensive nature of microwear analysis and limited spatial integrity of finds, previous studies have rarely applied the method alongside spatial analysis; often only a small number of flints are analysed which prevents the identification of any clear spatial patterning [36]. Studies have identified the spatial organisation of animal-related tasks such as antler/bone working and butchery at other Mesolithic settlement sites in Britain (Thatcham) [37] and mainland Europe (e.g. Årup, Vænget Nord, Lepenski Vir) [32,38,39]. However, very few microwear analysts consider the social implications for identifying activity zones within a structure. As such, many questions concerning the social structuring of domestic spaces remain unanswered. Interpretations often fall short of putting humans back into the picture, despite the role of individuals in the creation and working of tools [40,41]. Grøn’s work has been a key development in exploring the social dynamics of Mesolithic dwellings, using ethnographic case studies and social psychology [42–49]. Through ethnographic comparisons and tool typology, tools, namely microliths, and ‘dirty’ activities were used to identify male seating locations (e.g. tool knapping and arrowhead production), whereas female positions were identified through hearths or cooking materials [46,48,50]. When the social dimensions of tool use are interpreted from microwear analysis, ideas about the use of space and social structure are also lifted from the ethnographic record and used to interpret wear traces [e.g. 33].

These interpretations perpetuate gendered assumptions of Mesolithic dwellings without exploring alternative social structures [51,52]. It is within this broader context that a clear need exists for a study that implements microwear and spatial analyses to explore the social dimensions of activity areas, through a bottom-up approach. Tool use needs to be characterised prior to the interpretation of social aspects to the organisation of space. This is especially pertinent for Mesolithic settlements in Britain, which have not been sufficiently analysed and where the recovery of substantial quantities of well-preserved faunal remains alongside abundant and spatially discrete flints, hearths and hollows is rare [53–56]. Star Carr provides a unique opportunity to examine intra-site use of the earliest known Mesolithic structures in Britain, through a combination of new spatially plotted microwear data and previous technological, refitting and microwear data along with analysis of faunal remains and geochemistry of the areas associated with the structural features. By integrating these methods, it is possible to explore why people are deciding to repeatedly undertake activities in certain areas, and what this can tell us about community and the lives of people at the site.

### Star Carr

#### The site

**Fig 1:**
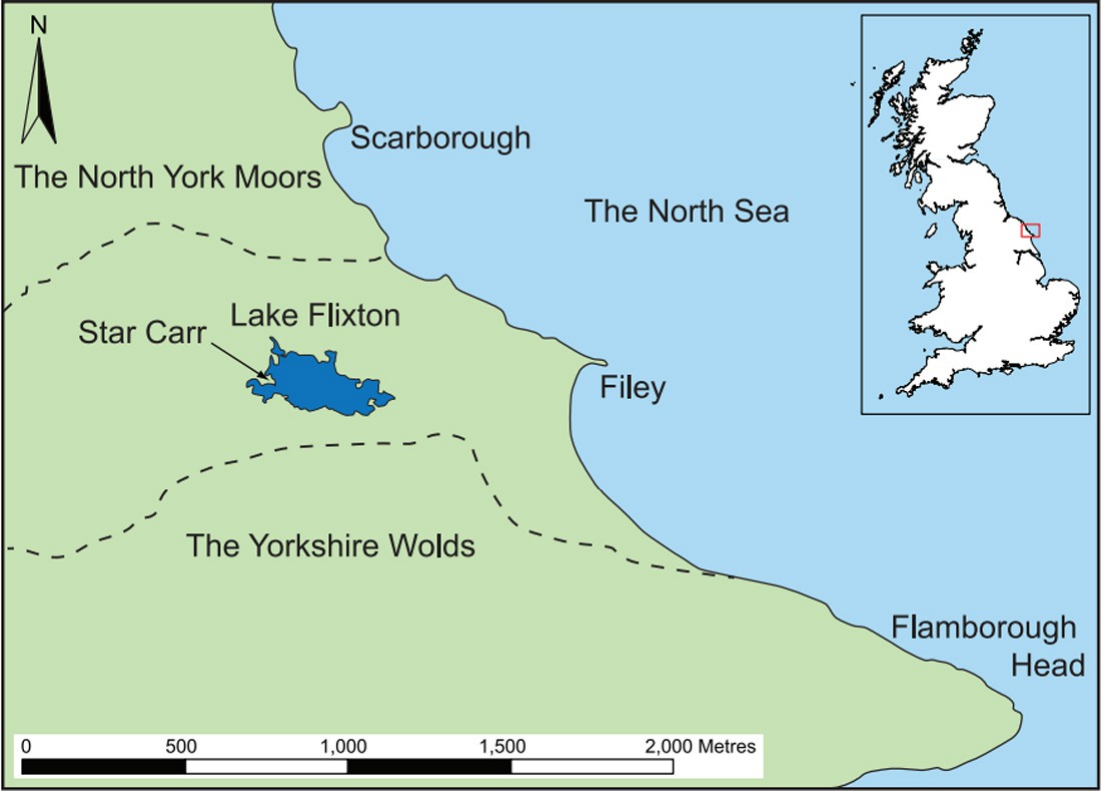
Location of Star Carr, North Yorkshire, UK. Republished from [60] under a CC license, with permission from the Star Carr Project, original copyright [2018].

Star Carr, an Early Mesolithic site (c. 9300–8500 cal BC) situated on a palaeolake in North Yorkshire (Fig 1), is renowned for its excellent preservation of organic remains [2]. Flint is the most abundant and consistently well-preserved archaeological material found at the site and has provided significant insights into the extent and nature of activities undertaken [57]. During excavations by the Star Carr Project (2004 to 2015), at least three post-built structures were identified [58]. These structures were found on the dryland—a raised area away from the lake edge where organic preservation was poor compared to the wetland; the associated faunal remains found were fragmented and highly degraded [58,59]. Cut features, such as postholes and hollows, were used to define the structures, along with associated deposits of flint and some faunal remains. These currently represent the earliest known post-built structures found in Britain [58].

Due to the excavation methods, which allowed for the recovery of all material, 41,820 pieces of knapped flint were excavated across the site and their locations recorded in 3D. Different analyses were applied with the aim of deepening understanding of the lithic assemblages, including: detailed technological analysis, refitting, residue analysis and microwear analysis [35,61,62]. Refitting analysis indicated that some retouched tools (e.g. burins, awls, truncations) were frequently moved from production areas to different locations across the site and cores were almost never found in the area where they were knapped [35,57]. The influence of human action on the final deposition of flint was recognised as significant and thus warrants consideration when interpreting spatially discrete results.

#### Previous microwear across the site

Microwear analysis previously undertaken by the Star Carr Project interpreted traces of use on 166 pieces (out of 220 analysed), relating to working antler, bone, fish, hide, meat, mineral, plant, wood and projectile impact traces [57]. Due to time limitations, a targeted sub-sampling approach was applied to flints found within key contexts (e.g. features, caches) and those of interest (pieces identified during technological analysis) [57]. The analysis aimed to assess the preservation of flint surfaces and any wear traces visible while also gaining insights into the range of tool-using activities at Star Carr and choices in tool use. A spatially scattered, targeted sub-sampling strategy was therefore appropriate [63]. Microwear results provided new information on how flints were manufactured, curated and deposited.

On the dryland, pieces at times appear to have been moved from their place of manufacture for use and through practices such as depositing in a midden, so it was important to explore these movements further in the context of the structures to assess intentionality in these individual actions [35]. Taphonomic issues of high rates of burning and abrasion from the depositional environment in the dryland were observed [57]. This contrasted with flint artefacts found in the wetland which were less frequently burnt and had been deposited in peat. Discrete areas of activity were inferred, such as tools used for plant working at the lake-edge, and a combination of refitting and microwear on a limited number of tools revealed that some were taken to structures for repair and storage [35]. Microwear analysis undertaken by Aimée Little (AL) provided small-scale insights into individual tool use across the site, with evidence that certain tools were potentially afforded value [35]. However, there remained questions regarding spatial patterns in tool use, specifically in relation to the structures and how activities were organised within these spaces.

#### Post-built structures

**Fig 2:**
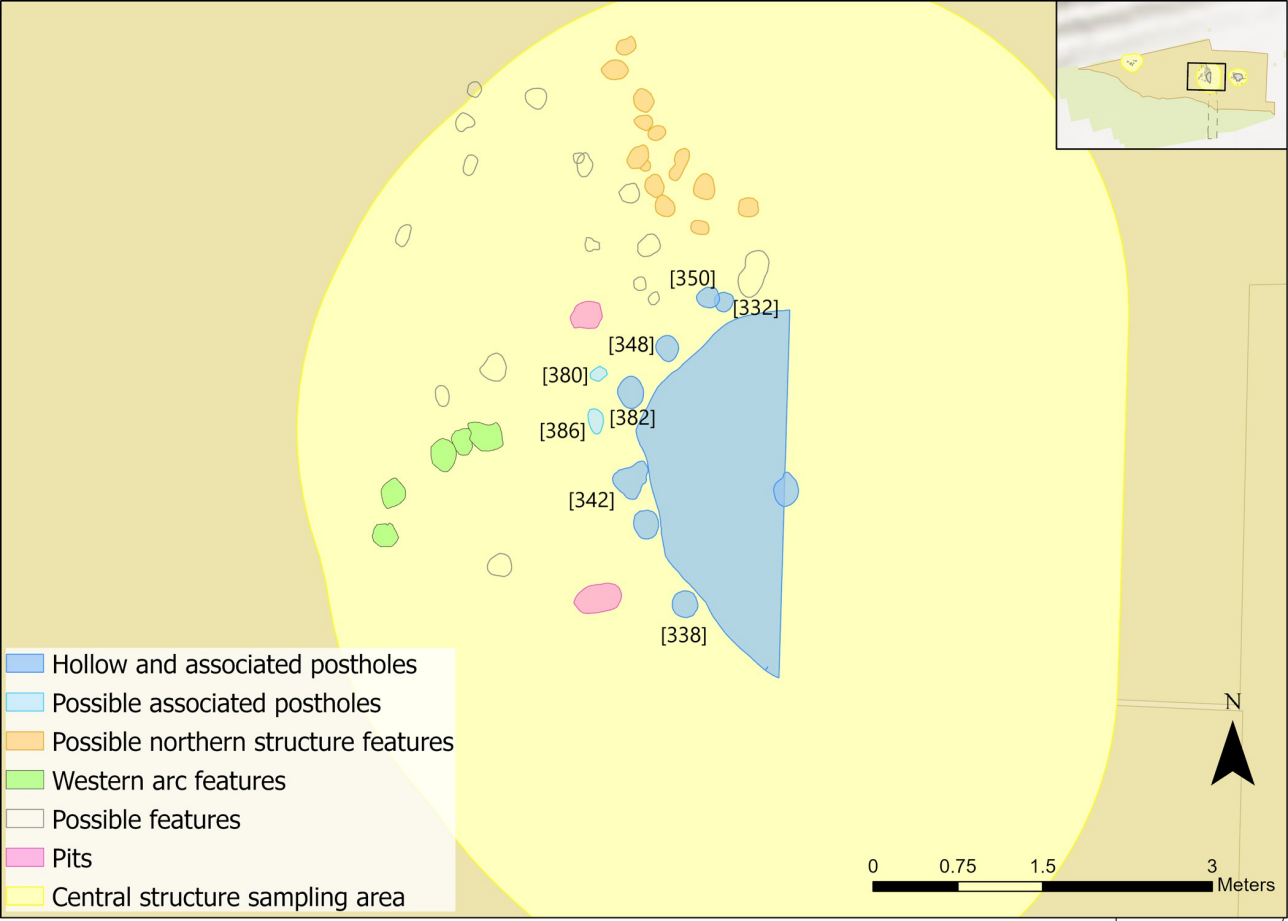
Plan of central structure features (after [58]).

**Fig 3:**
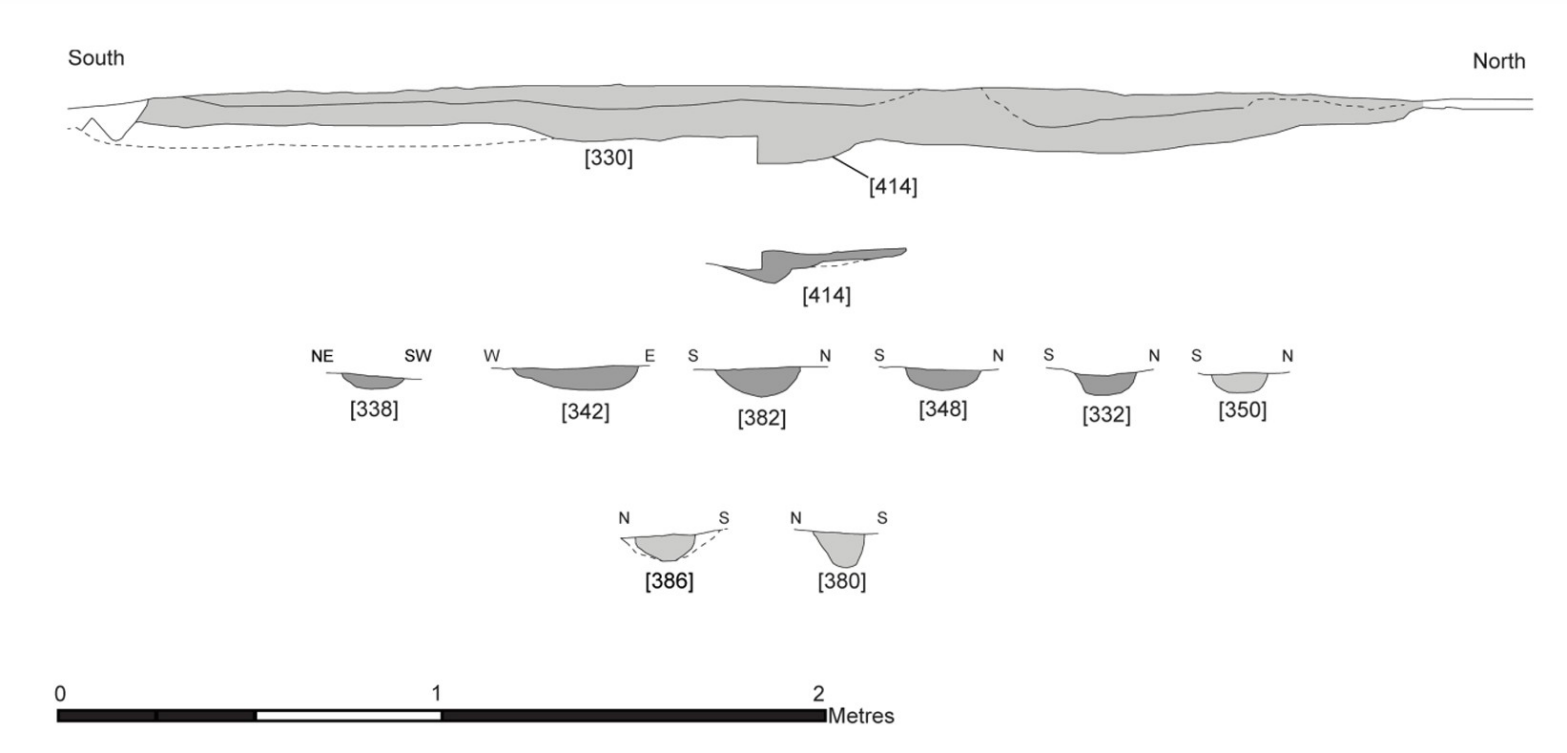
Feature profiles from the central structure. Republished from [58] under a CC license, with permission from the Star Carr Project, original copyright [2018].

The central structure was the earliest radiocarbon dated structure identified on the dryland, in use between 9300–9200 cal BC, and contained the largest quantity of associated postholes and pits [58,64]. A hollow, measuring 3.32m north to south, approximately 2.65m wide and 18cm deep, was surrounded by at least six postholes (Figs 2 and 3). It was truncated by previous excavations by the Vale of Pickering Research Trust (VPRT), meaning the full extent could not be uncovered [58]. Geochemical analysis of the soil elements in and around the structure indicated the presence of a wall [58,65]. Results further suggested that the inside of the structure was cleared of waste material or that different activities occurred inside the hollow compared to the surrounding areas [65].

**Fig 4:**
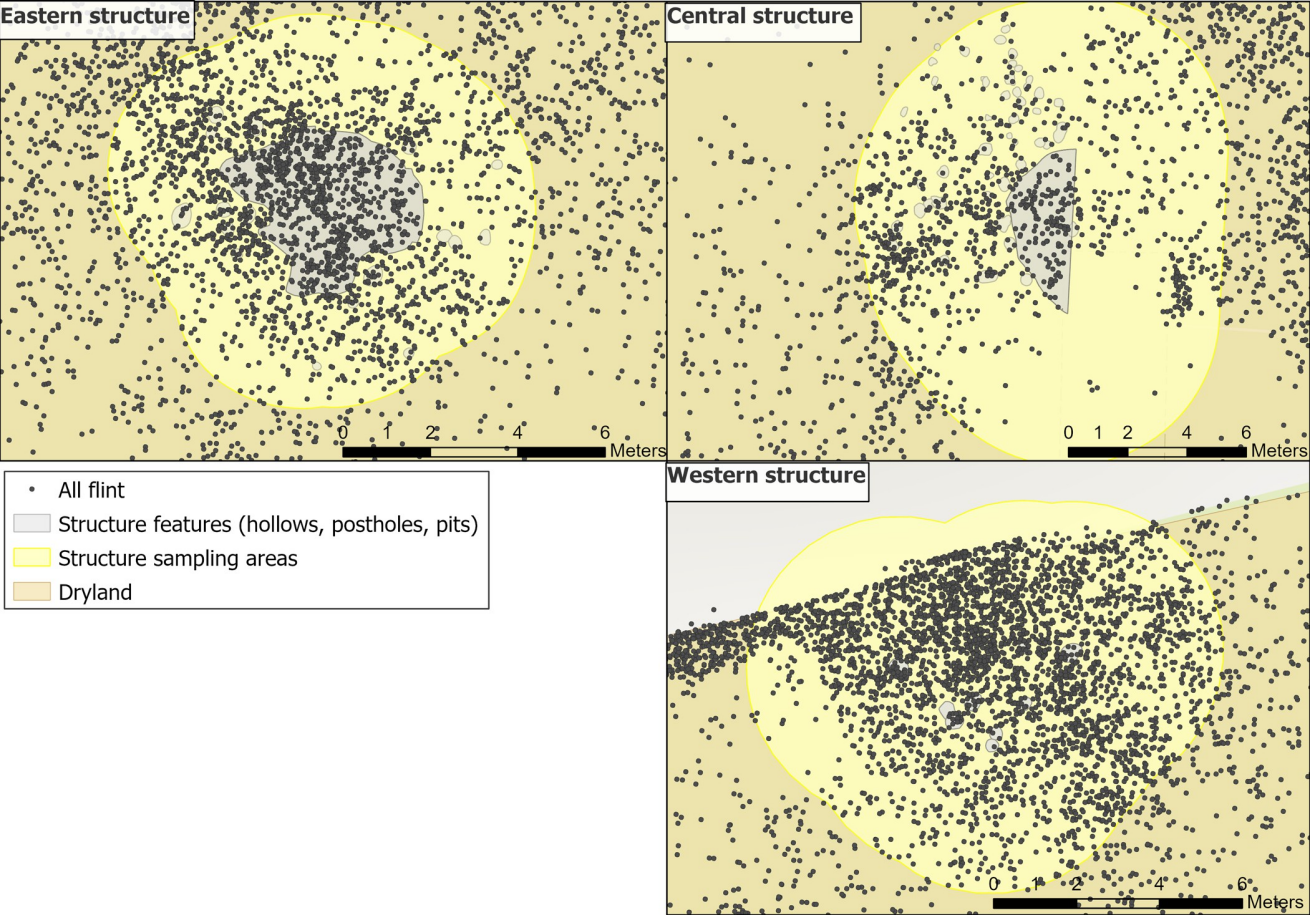
Composite image of all sampling areas and features with flint densities.

There was sparse material recovered from the hollow (12 pieces of animal bone, 407 pieces of flint, of which 4.2% were burnt); this, alongside the truncated hollow, has meant interpretations have understandably been left largely ambiguous, Fig 4 [35,58,66]. Additionally, radiocarbon dates show human activity dating to at least two different episodes: one earlier date c.9200 cal BC from the upper fill of the hollow and a later date c.8800 cal BC from post-hole [338] [64,67]. When the structure was in use, it was posited that it was either cleared regularly or was used differently to the other structures, which both contained higher flint densities [35]. It is possible that earlier inhabitants of the site may have used the central structure in such a way that fewer material traces were created to those seen in the other later structures [68–71].

Typological and microwear analysis of lithics helped elucidate some aspects of how the central structure was used. Discrete clusters of tool types were observed in the hollow: utilised blades to the west, three microliths to the north, and scrapers to the south-east (though some were excavated in the 1980s) [35]. Microwear analysis of a small sample revealed animal processing tasks took place within the hollow while refitting analysis demonstrated the movement of lithics and thus people in and around the area, with some activity likely postdating the structure’s use [35].

Additional features and post holes were found associated with the hollow (Fig 2) [58]. A lack of dateable materials and finds around these features meant interpretations could not be made beyond the shape and size of the features [58]. Due to limited evidence, they are included in the central area, rather than as stand-alone features. These post holes could relate to drying racks, perhaps for fish or meat, storage frames, wind breaks or frames for facilitating hide and/or other types of material processing [58]. To the west of the hollow there was a pit [336] which contained 49 pieces of worked flint and 24 pieces of burnt bone. To the south a possible pit [388] was identified which had no contents (Fig 2) [58].

**Fig 5:**
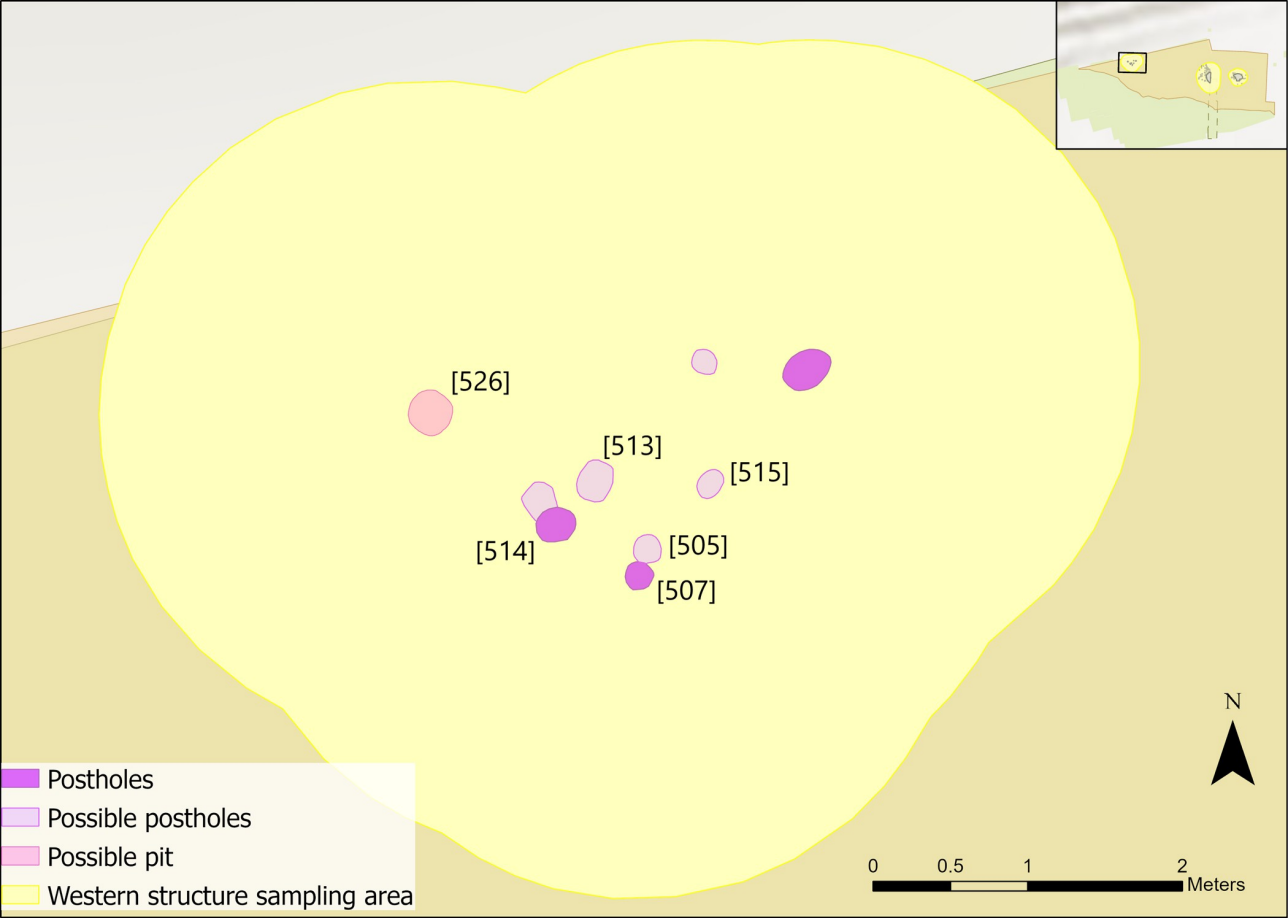
Plan of western structure features (after [58]).

**Fig 6:**
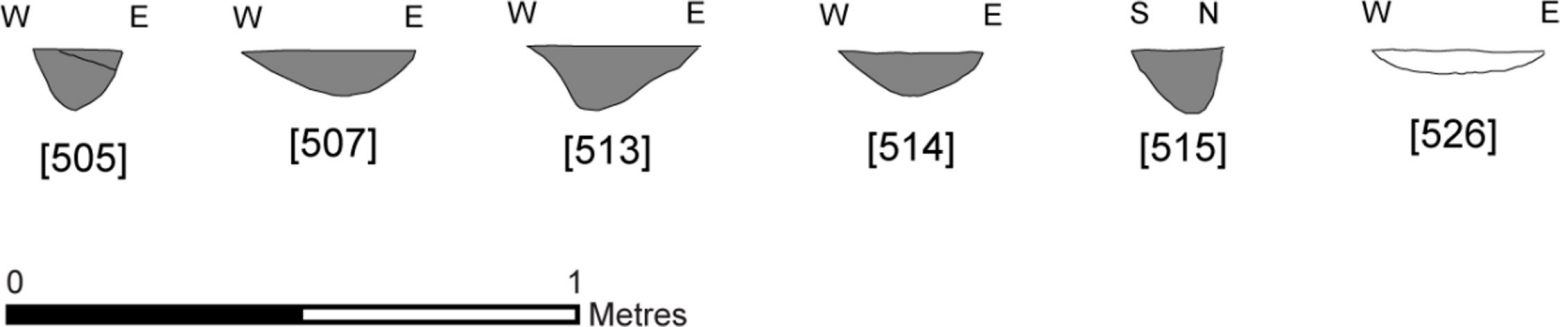
Feature profiles from the western structure. Republished from [58] under a CC license, with permission from the Star Carr Project, original copyright [2018].

The western structure consisted of nine small features without an associated hollow, making it the most tentatively identified structure [58]. Seven of these features were interpreted as likely postholes due to their profile and shape (Figs 5 and 6). The other two were identified as a possible post-hole [508] and a pit [526] [58]. Post-depositional processes significantly impacted the integrity of the features, thereby limiting interpretations of the structure’s composition and function. It is likely that the structure was built between 8945–8760 cal BC (95% probability), probably 8915–8895 (9% probability) or 8880–8795 (59% probability), therefore postdating the central structure [67].

Dense scatters of animal bone, antler and lithics were found in and around the western structure. In total, 5058 lithics are associated with the features, with c.35% burnt (Fig 4). Technological analysis identified 329 tools and 109 tool spalls; the highest density of flint and burnt material of all three structures [35]. A lack of clear spatial patterning in lithics and unburnt, likely *in situ* microdebitage, present conflicting interpretations of this area [35]. Due to limited refits, spatial patterns were assessed using tool type distribution. Apart from some discrete patterning of awls, tool types were mixed. Previous microwear results by AL indicated that craft-based activities (e.g. bone, antler and mineral working) were undertaken to the south of the features and traces of bone working observed on flint tools seemed to correlate with densities of animal bone found nearby, suggesting *in situ* bone working activity in and around this area [35,59]. This evidence, together with high levels of burning observed within the perimeter of the postholes, was used to suggest that either: 1) the structure had burnt down, 2) or it was a midden on top of a previous structure [35,72].

Flint densities are concentrated to north of the postholes (Fig 4), so it is possible that the excavated features are not complete. A structure may have extended beyond the excavated area to the north, with the possibility of further postholes remaining unidentified. The high levels of burnt lithics recovered from this area may suggest that waste materials, including burnt flint, were deposited together within this possible structure [64].

**Fig 7:**
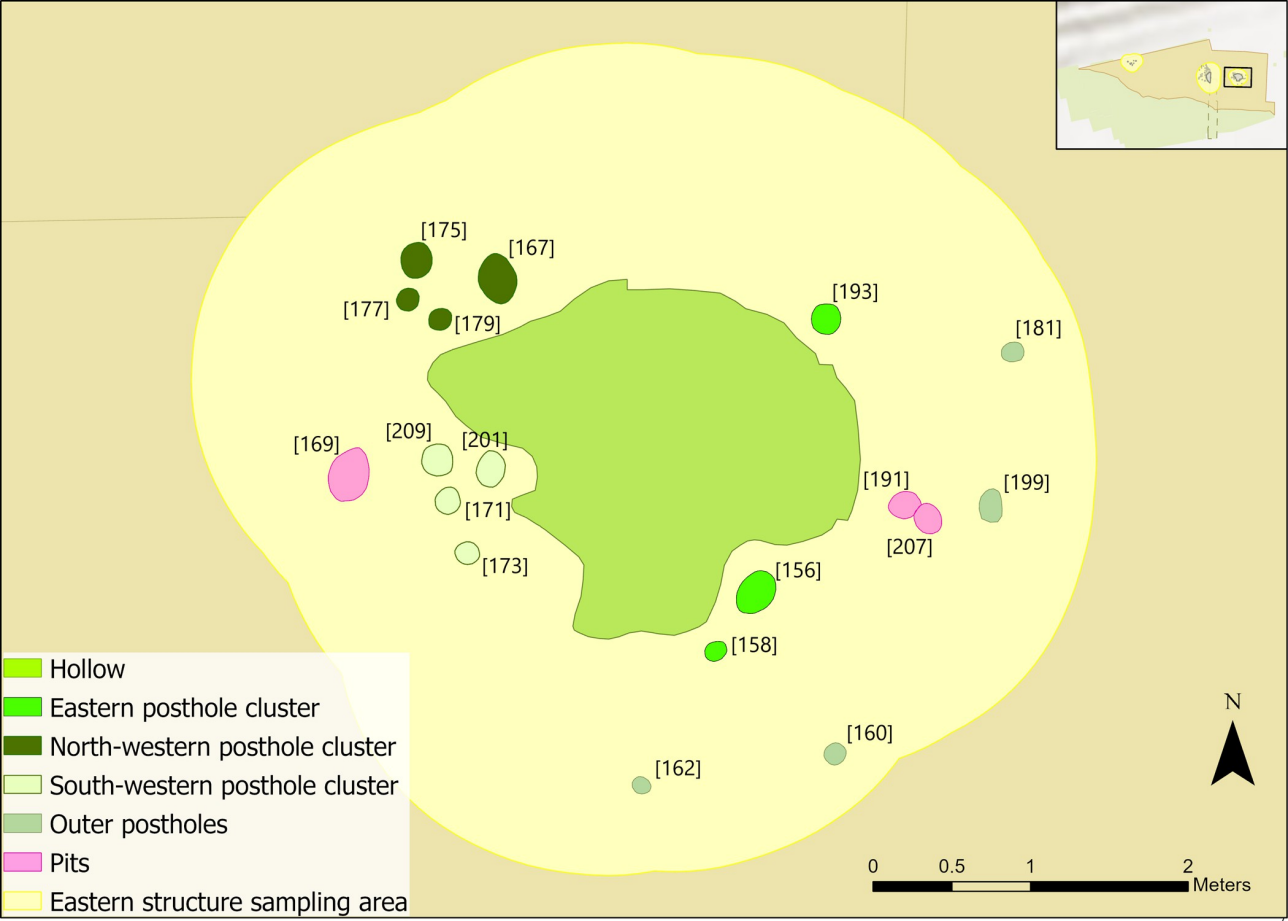
Plan of eastern structure features (after [58]).

**Fig 8:**
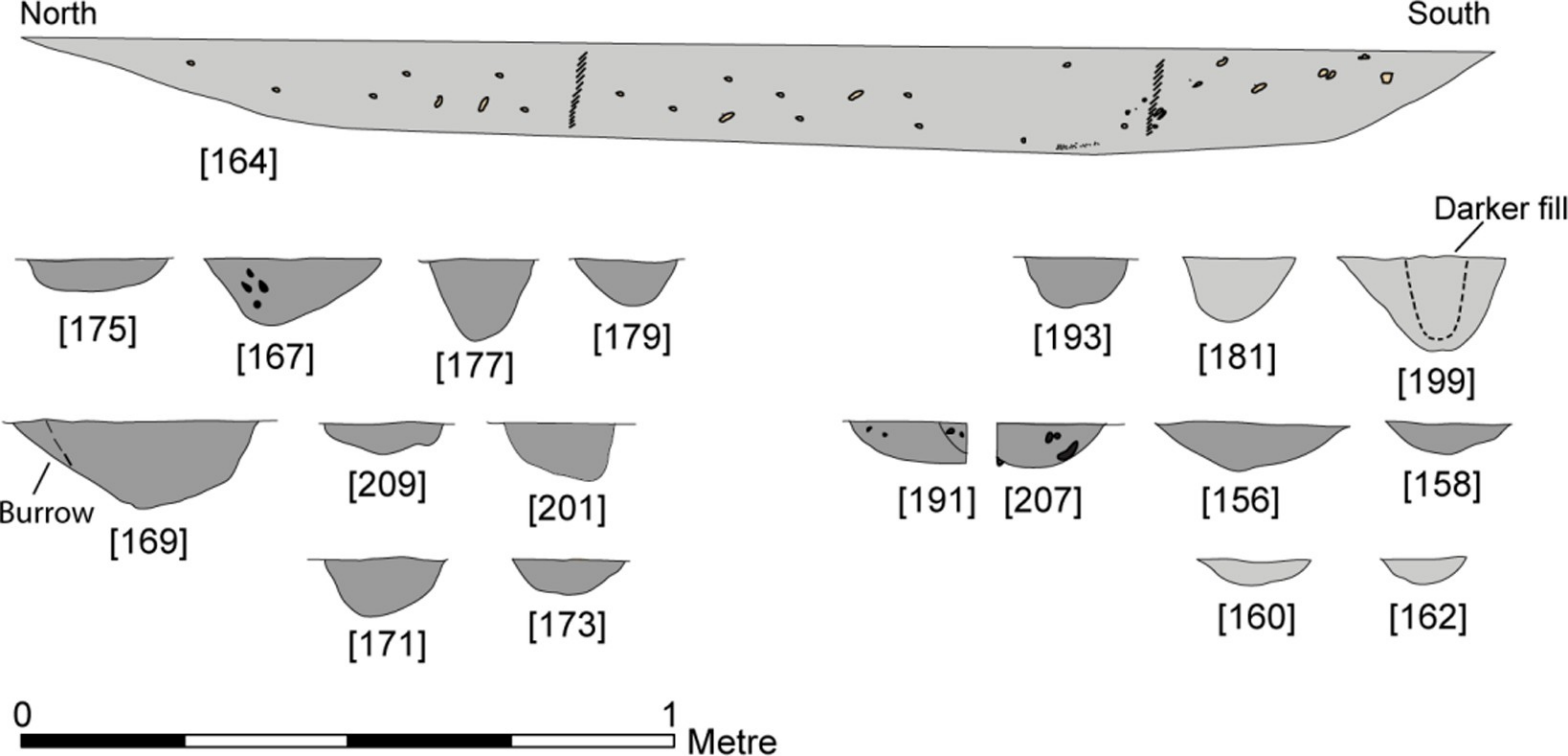
Feature profiles from the eastern structure. Republished from [58] under a CC license, with permission from the Star Carr Project, original copyright [2018].

The eastern structure comprised a hollow measuring roughly 20cm deep and at least 2.8m wide surrounded by postholes (Figs 7 and 8) [58]. Radiocarbon dates suggest that it was constructed at a similar time to the western structure [67]. Most features (15) were interpreted as postholes, holding largely upright posts, consisting of an outer and inner arc, with two clusters to the west (Fig 7) [58]. Three small pits were also found [58]. The hollow had two fills, a lower one containing high organic content and an upper fill where most lithics were recovered [58]. Micromorphological analysis suggested a high organic content in the lower fill which was probably resulting from a basal layer of plant material. [58,71,73]. No samples were taken for geochemical analysis, so interpretations of the structure relied on flints and faunal remains.

Significant quantities of lithic material were found in the eastern structure, numbering 1921 pieces of which 23.3% were burnt (Fig 3). Most lithics were found in the upper fill, located above the plant layer, which contained very few artefacts. This has been interpreted as a possible organic matting, such as reeds or bark, which may have prevented most flint from moving down the soil profile [58]. From refitting and metrical analysis, lithic material was interpreted as being moved from within the structure to adjacent middens over a significant amount of time. It was noted that larger pieces were cleared from within the structure whilst smaller, more fragmented material resulting from *in situ* knapping were left inside the hollow [35]. Refitting indicated that particular tools were manufactured inside the structure (burins and scrapers) whilst composite tools (e.g. hafted axes and microliths) were likely stored and maintained in this area. Microwear analysis undertaken by AL on six tools showed different tasks undertaken inside the hollow, while refitting showed resharpening of some artefacts within the structure indicating that a tool kit was stored here and suggesting a ‘household level of ownership of certain tools’ [57, p.533]. Food processing, eating, tool maintenance, sporadic tool use for craft activities were all inferred as taking place in the structure [71,72].

## Materials and methods

### Materials

In total, 341 flints were analysed as part of this programme of study: 148 from the eastern structure, 102 from the central structure and 91 from the western structure. All pieces included in the analysis had x, y and z coordinates and feature hollows had x and y coordinates. Due to bioturbation it was not possible to establish a stratigraphy of discrete flint scatters within the hollows.

Analysed lithics were generally in a good condition, but PDSM (post-depositional surface modification) was still observed on a significant proportion of the assemblage (54% based on samples from across the site) [62,74]. This includes surface patination (white and gloss); trampling; post-excavation marks; iron oxide deposits and staining. The dryland depositional context at Star Carr meant that a large proportion of flint had iron oxide staining over some or, in some cases, most of the flint edge [74,75].

### Methods

#### Microwear

Two phases of sampling were used to select flint for microwear analysis. The first phase involved plotting flint in GIS to initially define areas of interest, before sub-sampling those areas for microwear analysis. For the second phase that targeted the eastern structure, an initial scan using low power magnification (10x) was used to assess signs of use on flint from a small defined area, and those with possible use were analysed fully with higher power magnification (20x). The eastern structure was selected for further analysis because flint pieces were better preserved (levels of PDSM and iron oxide staining) and archaeological features were the most complete, with some associated faunal remains. An absence of use traces was noted on pieces that were assessed. All burnt flints were excluded from the analysis as it can impact the preservation of microwear traces [76,77].

A standard cleaning procedure was developed using an Ultrawave U300 ultrasonic cleaning bath to remove residual dirt and finger grease on all flints with washing soap [78,79]. Prior to analysis, artefacts were also cleaned with an ethanol-soaked cotton pad. This was repeated throughout, using cotton buds to spot clean specific areas whilst the flint was mounted on the stage.

Microwear analysis was undertaken in the Imaging and Wear Analysis (IAWA) Lab within the Centre for Artefacts and Materials Analysis (CAMA), University of York. A low-power Olympus SZ61 microscope (between 6.7x to 45x magnification, using 10x eyepieces) with an Olympus LC30 camera was used, along with a high-power reflected light Olympus BX53M (10x and 20x magnification, using 10x eyepieces) with an Olympus DP74 camera attached to a desktop computer. High quality images of microwear polish were taken during analysis using stacking software on STREAM (Olympus microscope imaging programme) to ensure that most aspects of the flint surface were in focus. Microwear recording forms were used to record information about each piece, these were based on those used by the Leiden Laboratory for Artefact Studies [63,79]. A reference collection produced by an experienced experimental archaeologist was used to aid interpretations of microwear traces. Reference material replicated the types of tasks—working on contact materials—that previous studies have shown were likely to have occurred at Star Carr. If the contact material could not be interpreted, the hardness of material and directionality was noted, where possible. Hardness of contact material does not provide the level of detail given by a specific contact material (e.g. plant); however, it can help to reduce the range of possible materials worked.

#### Geographical Information System (GIS)

**Fig 9:**
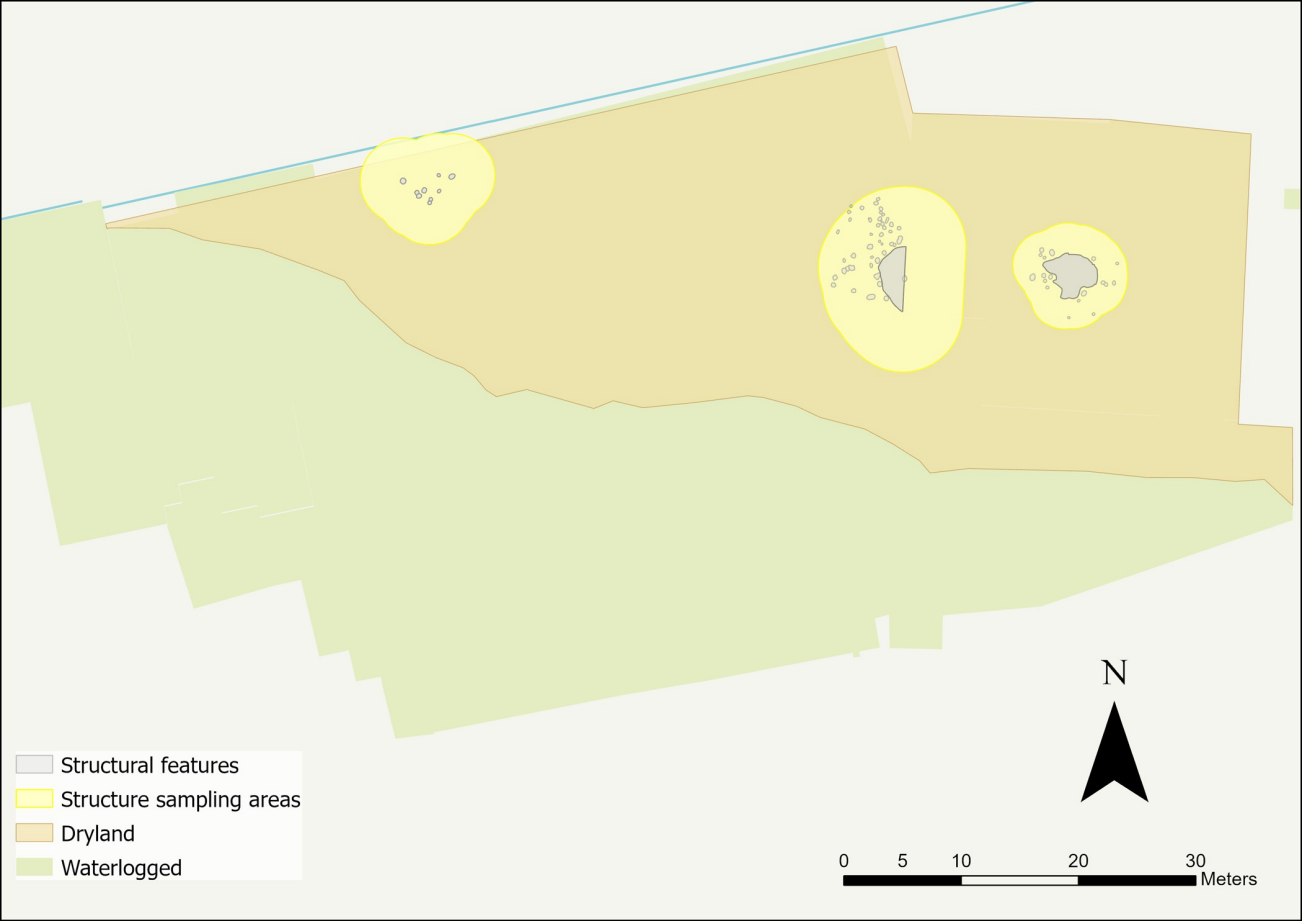
Plan of all sampling areas for each structure in yellow and their associated features.

The use of GIS was integral for both sampling and interrogation of microwear results. One of the aims of this analysis was to explore the spatial extent of the structures, as it is possible that they would have covered a larger area than mapped by postholes alone (e.g. roof rafters were likely to have tapered down to the ground and may have extended beyond the postholes). Therefore, a larger periphery around each structure was established for sampling to enable comparisons of results from both within and immediately surrounding the structures. To create a periphery, Optimised Hot Spot Analysis was used in GIS to visualise flint densities. Locations of associated features (e.g. post holes, pits) were then mapped alongside the hot spot analysis results to establish a sampling radius surrounding each structure (Fig 9): 1.5 metres for the eastern structure, 2 metres for the western structure and 3 metres for the central structure.

Once results had been obtained from microwear analysis, they were inputted into GIS and spatially plotted to examine patterns in tool use across the sampling areas. All results (including tools interpreted as not used or those inferred as used on indeterminate materials) were mapped. Where flints have been noted as not used, this means that no interpretable wear traces were observed during analysis. Pieces may still have been used as tools, but the action resulted in no identifiable microwear traces. Only those interpreted as used on a specific contact material are presented and discussed in this paper as they provide a clear picture of the types of activities undertaken.

## Results

### Overview

The following section presents a summary of the new microwear results from each structure. Rates of tool use, including the range of materials inferred, are stated alongside the frequency of PDSM observed. Where more than one type of contact material is noted, this generally denotes microwear found at different locations across the tool’s edge, for example hafting traces and meat microwear traces, rather than overlapping polish in the same area. An exception to this is where additives have been interpreted alongside hide working (i.e. tools with hide and mineral working traces). A selection of micrographs is also presented for each structure, to evidence the range of polishes observed. Results of all analysed pieces can be found in S1 Table.

### Central structure

Across the central structure area 102 lithics were analysed, 28 of which were located in the central hollow area (27% of pieces analysed). Signs of use were observed on 47 pieces (46%) with 55 interpreted as not used (54%). Lithic artefacts were sub-sampled based on tool type and spatial location; they were selected from across the study area to ensure that those associated with different features were analysed. An even spread of pieces across the area was not always possible as some flint could not be located in the archive. Where a cluster of a particular tool type was identified, if possible, several were analysed to explore potential links in flint use in certain areas.

Artefacts sampled had relatively high rates of PDSM, 71% of pieces showed at least one type. Iron oxide staining was observed on 43, 22 had flat dull smoothing, 10 showed metallic striations, and three had surface patination. Where polish was observed, iron oxide staining rarely prevented an interpretation of contact material. In the few cases where PDSM impacted interpretations, hardness of material and directionality was noted in lieu of a specific contact material.

**Fig 10:**
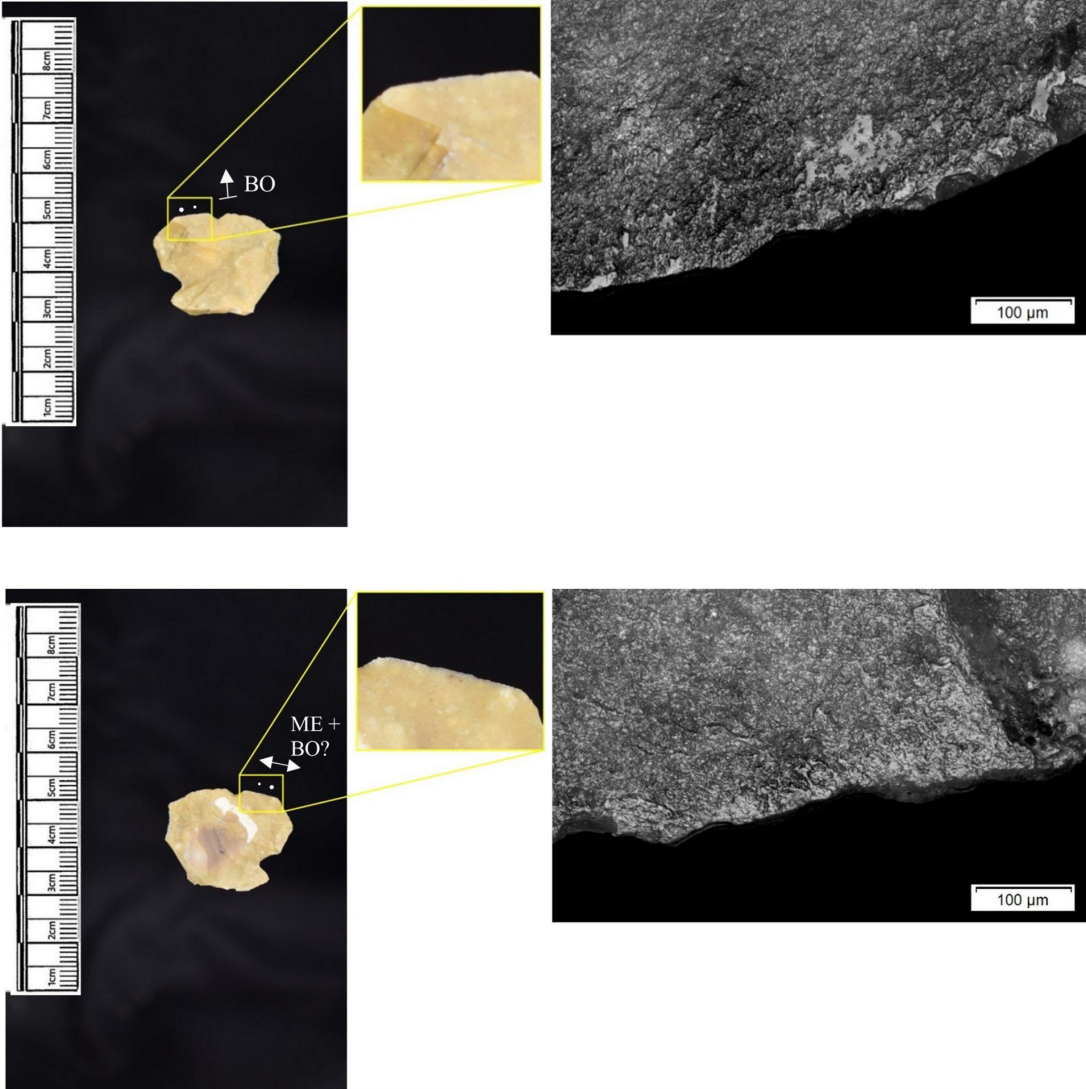
SC 96663, a flake interpreted as used to scrape bone and cut meat and bone, 200x magnification.

**Fig 11:**
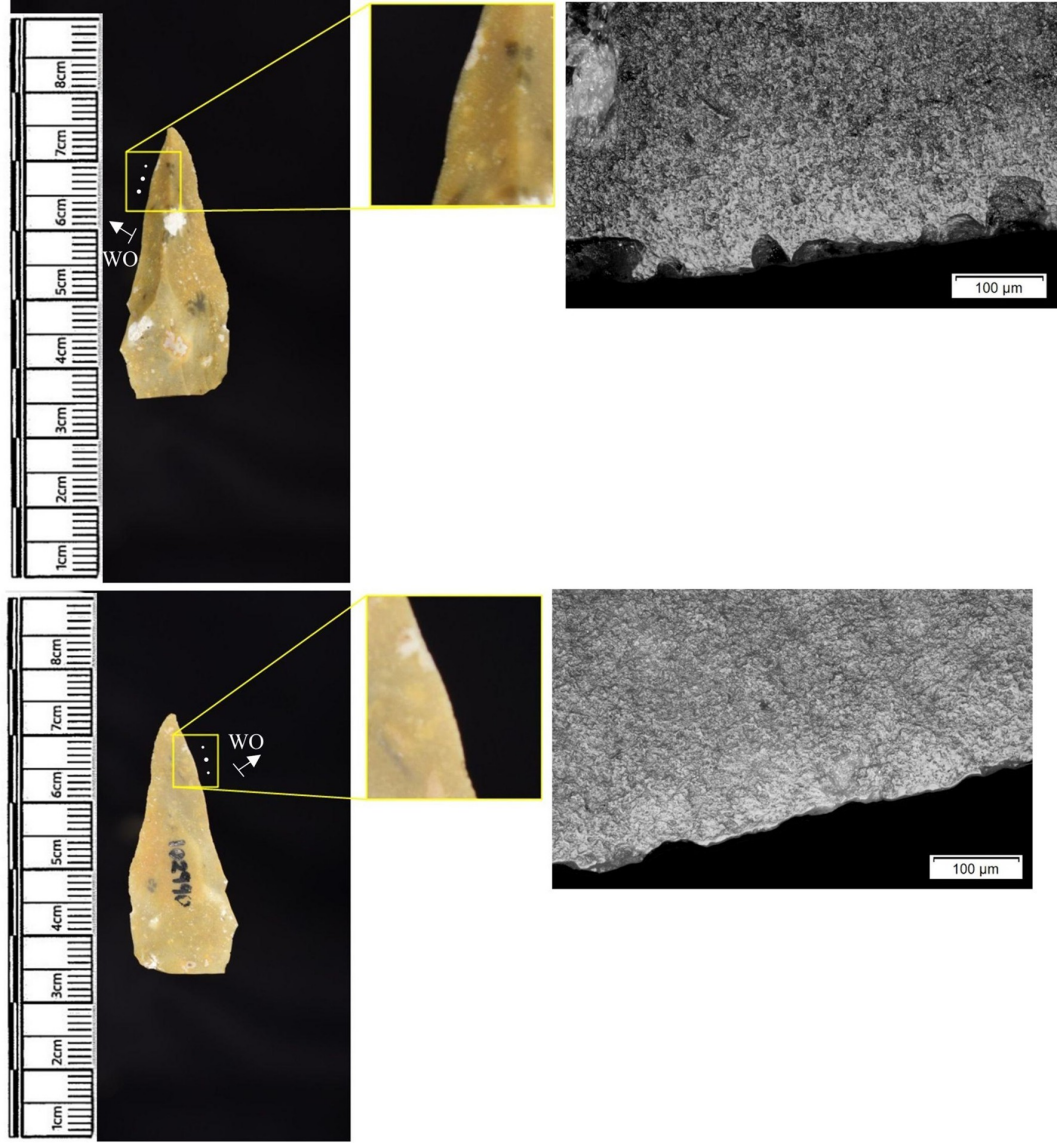
SC 102990, a bladelet showing developed polish from scraping soft or green wood, 200x magnification.

**Fig 12:**
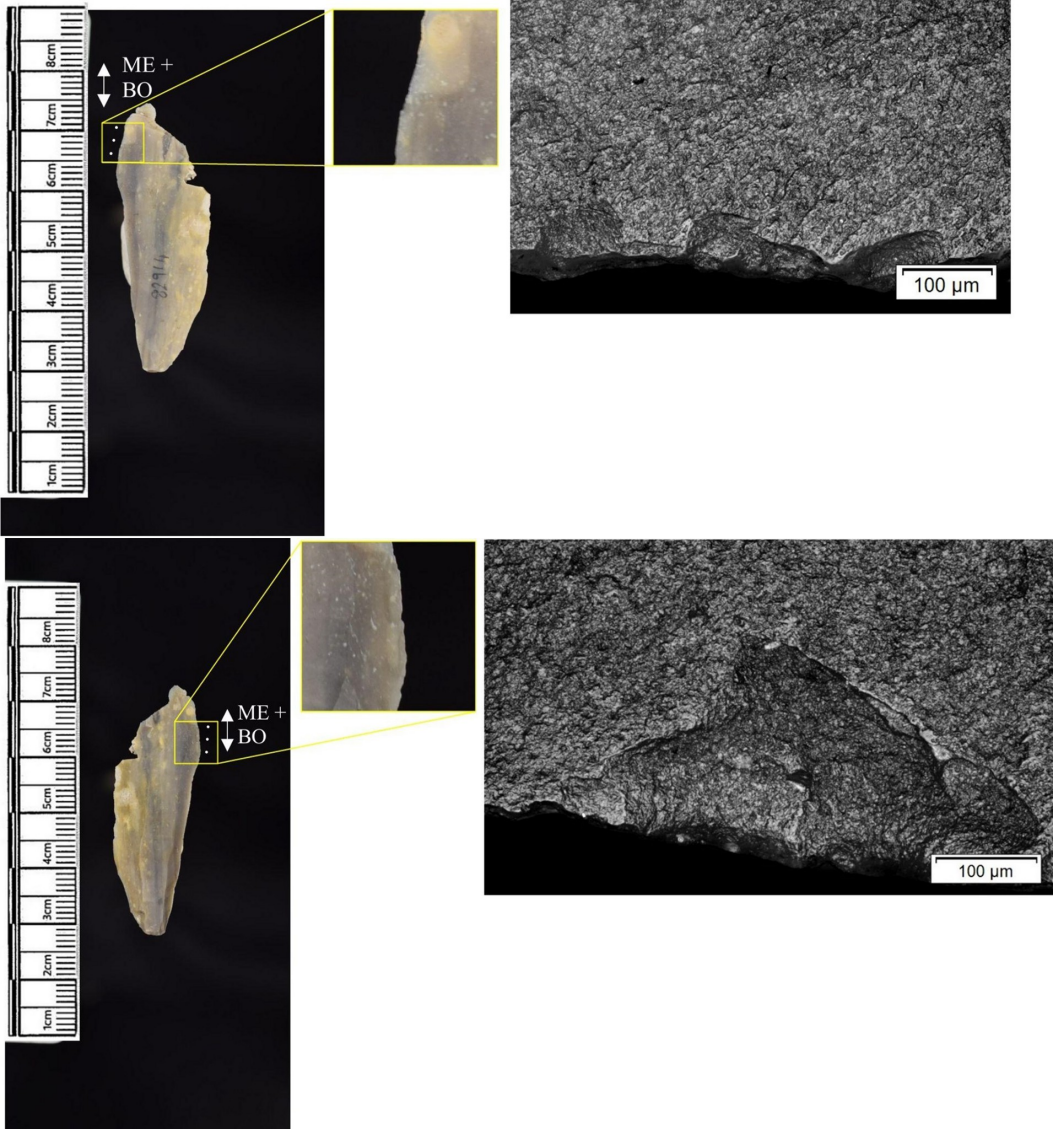
SC 82914, a bladelet interpreted as used to cut meat and bone, 200x magnification. The polish observed was not well developed on either aspect.

**Fig 13:**
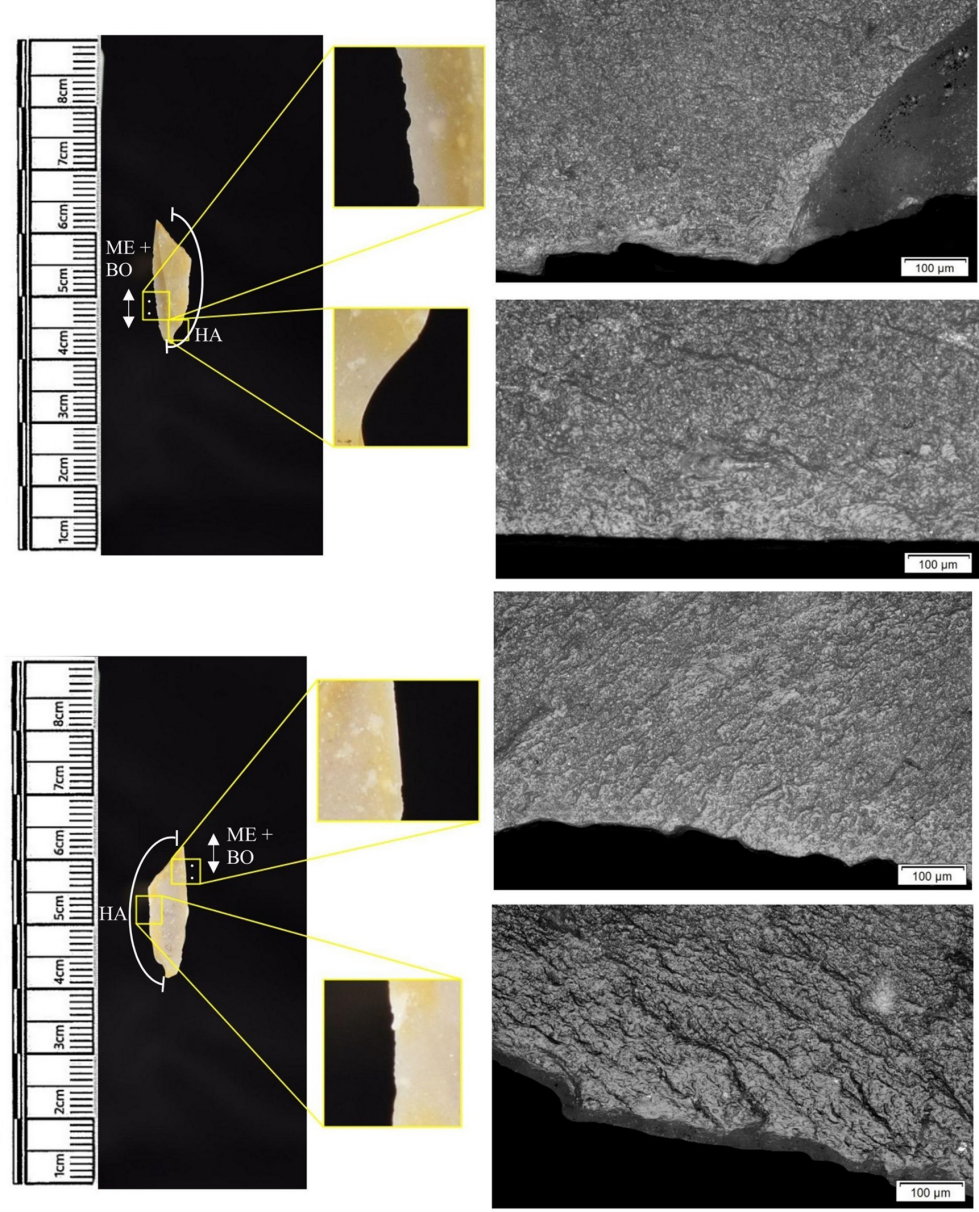
SC 108397, a microlith interpreted as hafted and used longitudinally on meat and bone (unclear whether used as a projectile due to absence of MLITs), 200x magnification.

The most frequently observed microwear traces from the central structure were from working bone (n = 10 –Fig 10), followed by wood (n = 6 –Fig 11) and hide (6). Plant (n = 3), fish (n = 3), meat (n = 1 –Fig 12), antler (n = 1) working and the use of projectiles (n = 2 –Fig 13) were also interpreted (Table 1).

**Table 1:** Summary of tool use from the central structure.

Primary contact material	Secondary contact material	Motion of use	Number
Antler		Transverse	1
Bone		Longitudinal	5
Bone	Meat	Transverse; longitudinal	3
Bone	Hide	Transverse	2
Fish		Transverse; longitudinal	3
Hide		Transverse	2
Hide	Soft mineral	Transverse	3
Hide	?Wood	Transverse	1
Meat	Bone	Longitudinal	1
Projectile	Meat + bone	Longitudinal (hafted)	1
Projectile	Medium indeterminate	Longitudinal (hafted)	1
Plant (inc. plant/soft wood)		Transverse; longitudinal	3
Wood		Transverse; longitudinal	6

### Western structure

In total, 88 artefacts were analysed from the western structure area, 35 (40%) were located within a triangular area created by the features. Flints located in close proximity to post holes were prioritised for analysis and those found in the wider area were also sampled to capture possible differences in use. Overall, 36 pieces showed no signs of use (41%), making the rate of used flints 59%. If a cluster of the same tool type was observed, a selection was analysed, where possible.

Rates of PDSM were high: 72% of pieces showed at least one type. Iron oxide staining (30) and flat dull smoothing (39) were the most frequently observed, with 13 flints displaying metallic striations and five had surface patination. Levels of flat dull smoothing were the highest compared to the other structures. This type of PDSM often occurs from trampling or contact with other flints, suggesting that the depositional conditions in the structure differed from the other two structures [80]. Generally, PDSM did not prevent the interpretation of microwear traces as 33 tools were interpreted as used despite the presence of PDSM.

**Fig 14:**
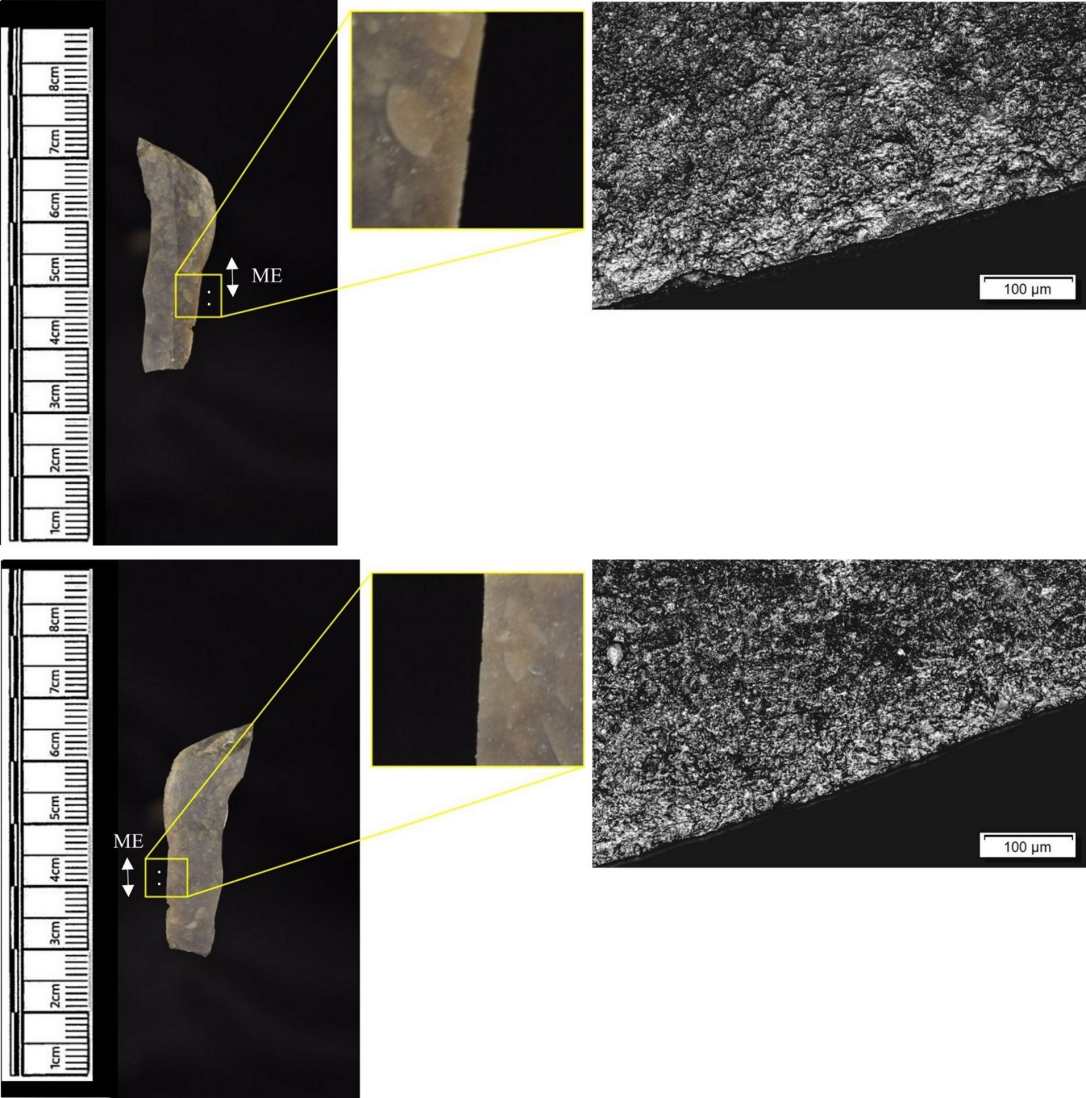
SC 109673, a burin spall interpreted as used to cut meat, 200x magnification.

**Fig 15:**
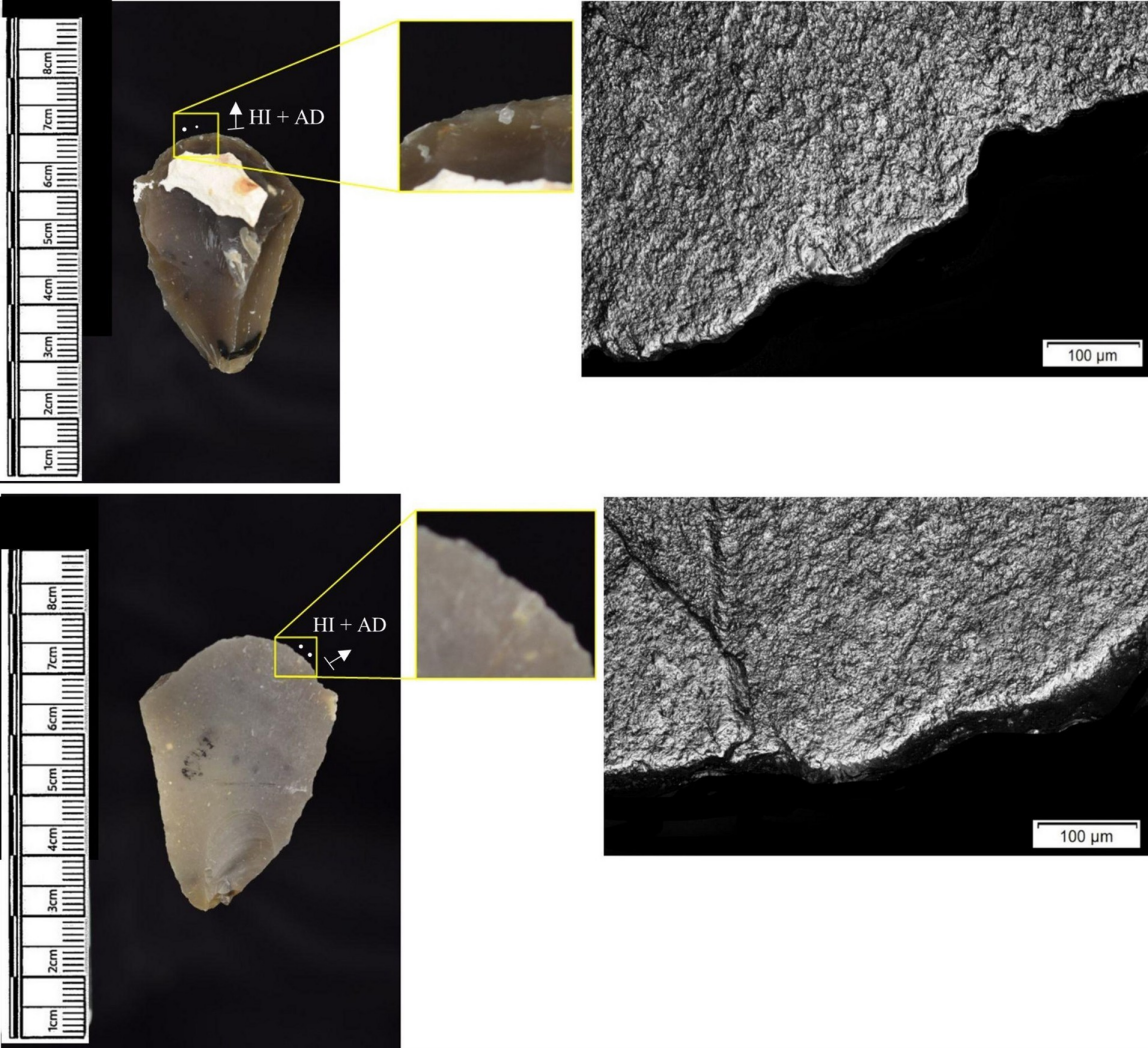
SC 95428, a scraper used to scrape dry hide with an additive, likely mineral due to the flat areas of the polish, 200x magnification. The polish is particularly well developed on the ventral aspect.

**Fig 16:**
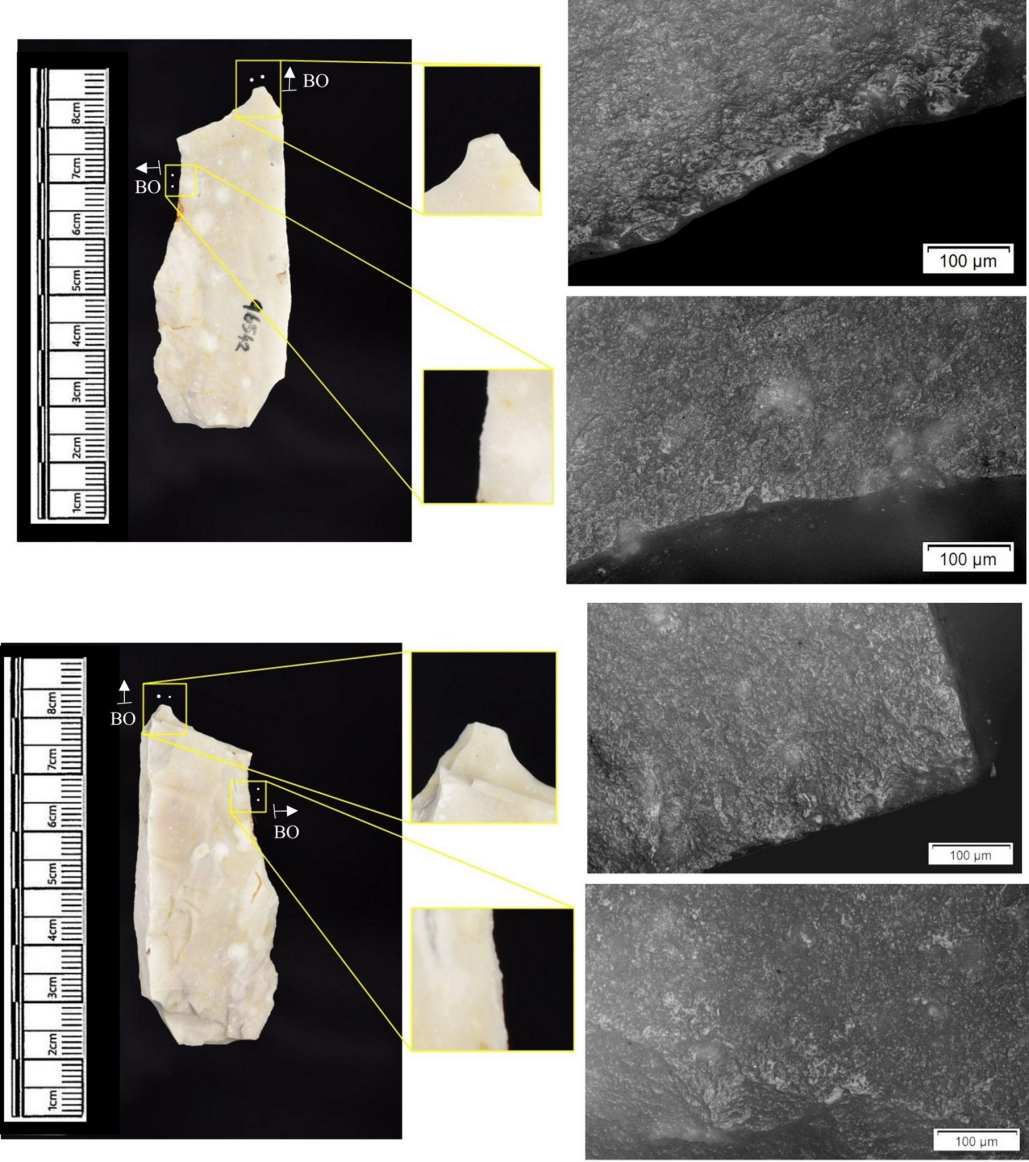
SC 96542, a blade interpreted as used to engrave and scrape bone, 200x magnification.

**Fig 17:**
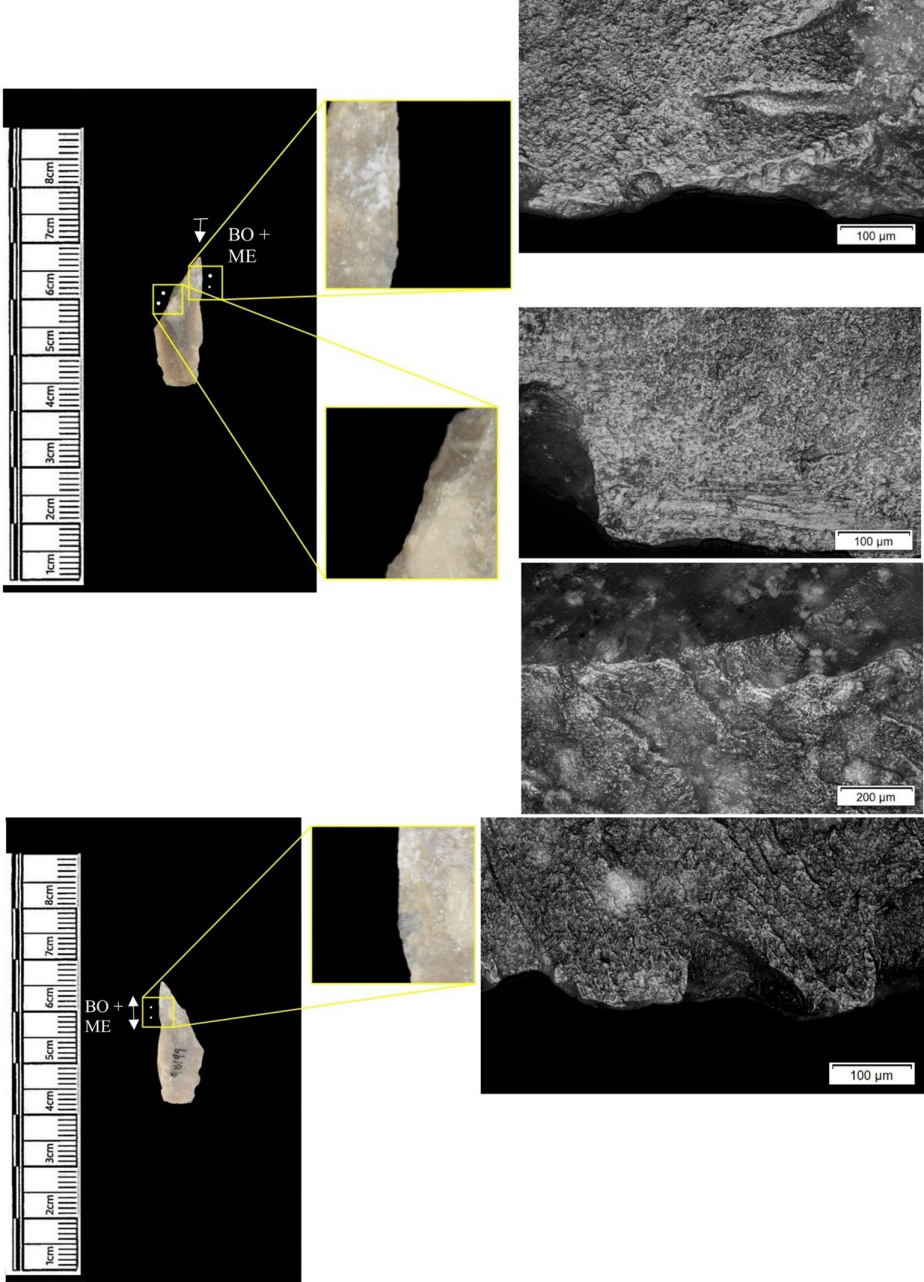
SC 98199, a microlith interpreted as a projectile based on faint MLITs on the dorsal aspect, as seen in the bottom two micrographs from the dorsal aspect. The projectile displayed contact with bone and meat, 200x magnification and 100x magnification for the lower dorsal image.

**Fig 18:**
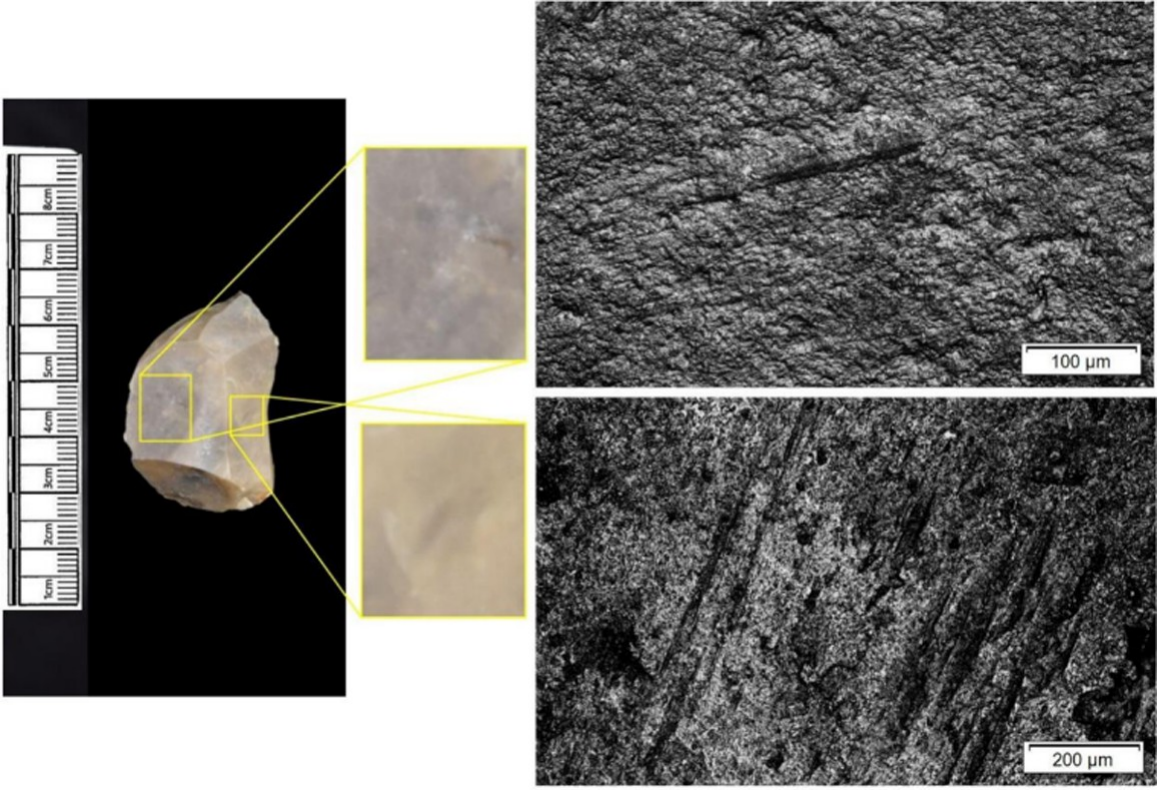
SC 102669, a core interpreted as used as a strike-a-light in two areas, 100x and 200x magnification. There was no polish observed on the dorsal.

**Fig 19:**
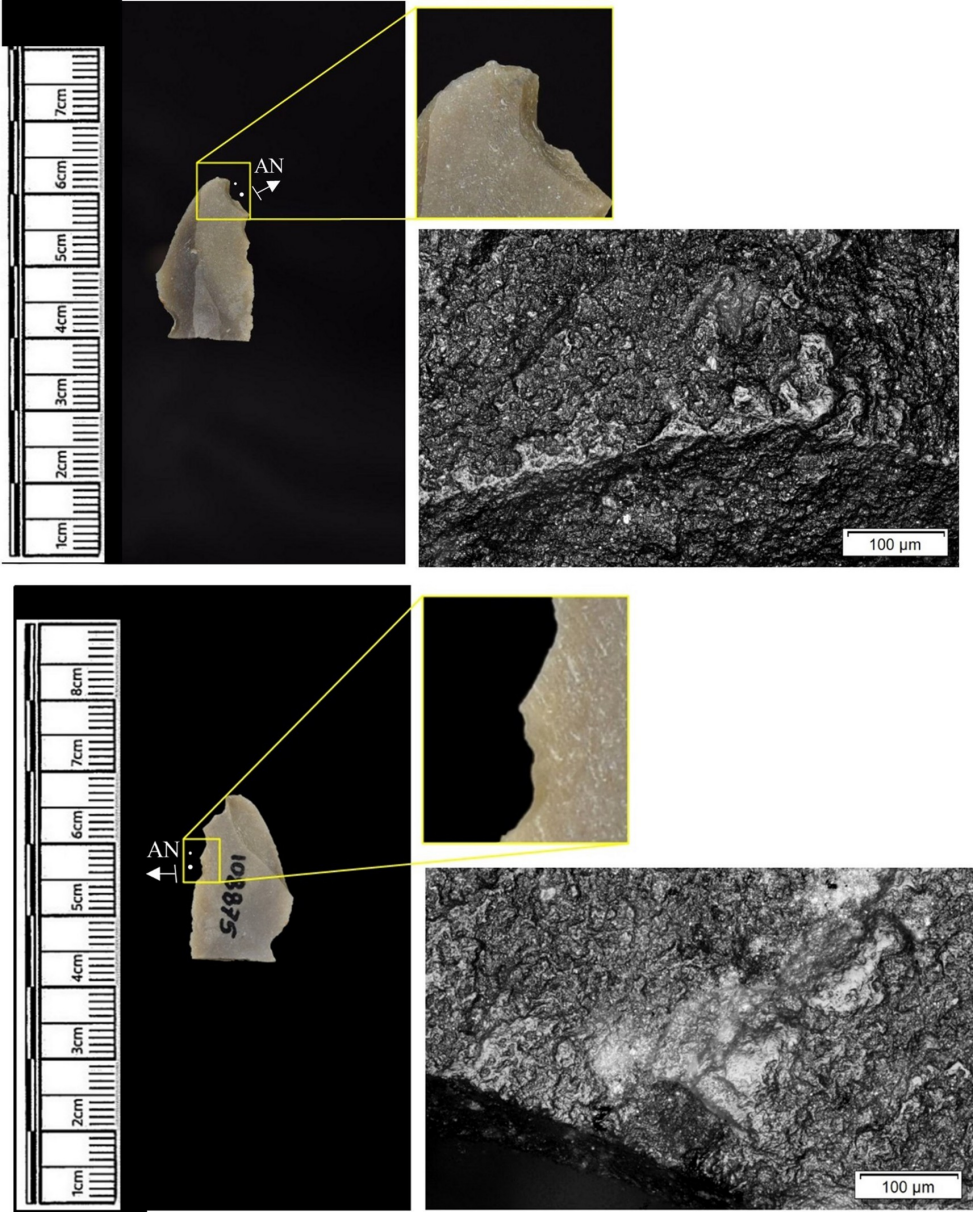
SC 108875, a microburin interpreted as used to groove and plane antler, 200x magnification.

Materials worked within the western structure were diverse (Table 2). Meat was most frequently observed (n = 8 –Fig 14), followed by: hide (n = 7 –Fig 15), bone (n = 5 –Fig 16), wood (n = 5), projectile impact (n = 2 –Fig 17), mineral (n = 2 –Fig 18), fish (n = 2), plant (n = 1) and antler (n = 1 –Fig 19).

**Table 2:** Summary of tool use in the western structure.

Primary contact material	Secondary contact material	Motion of use	Number
Antler		Transverse	1
Bone		Transverse	4
Bone	Meat	Transverse; longitudinal	1
Fish		Transverse; longitudinal	2
Hide		Transverse	3
Hide	Soft mineral	Transverse	4
Meat		Longitudinal	4
Meat	Bone	Longitudinal	3
Meat	Hide	Transverse; longitudinal	1
Projectile impact	Bone and meat	Longitudinal (hafted)	2
Mineral (strike-a-light)		Striking	2
Plant (inc. plant/soft wood)		Longitudinal	1
Wood		Transverse; longitudinal	5

### Eastern structure

**Fig 20:**
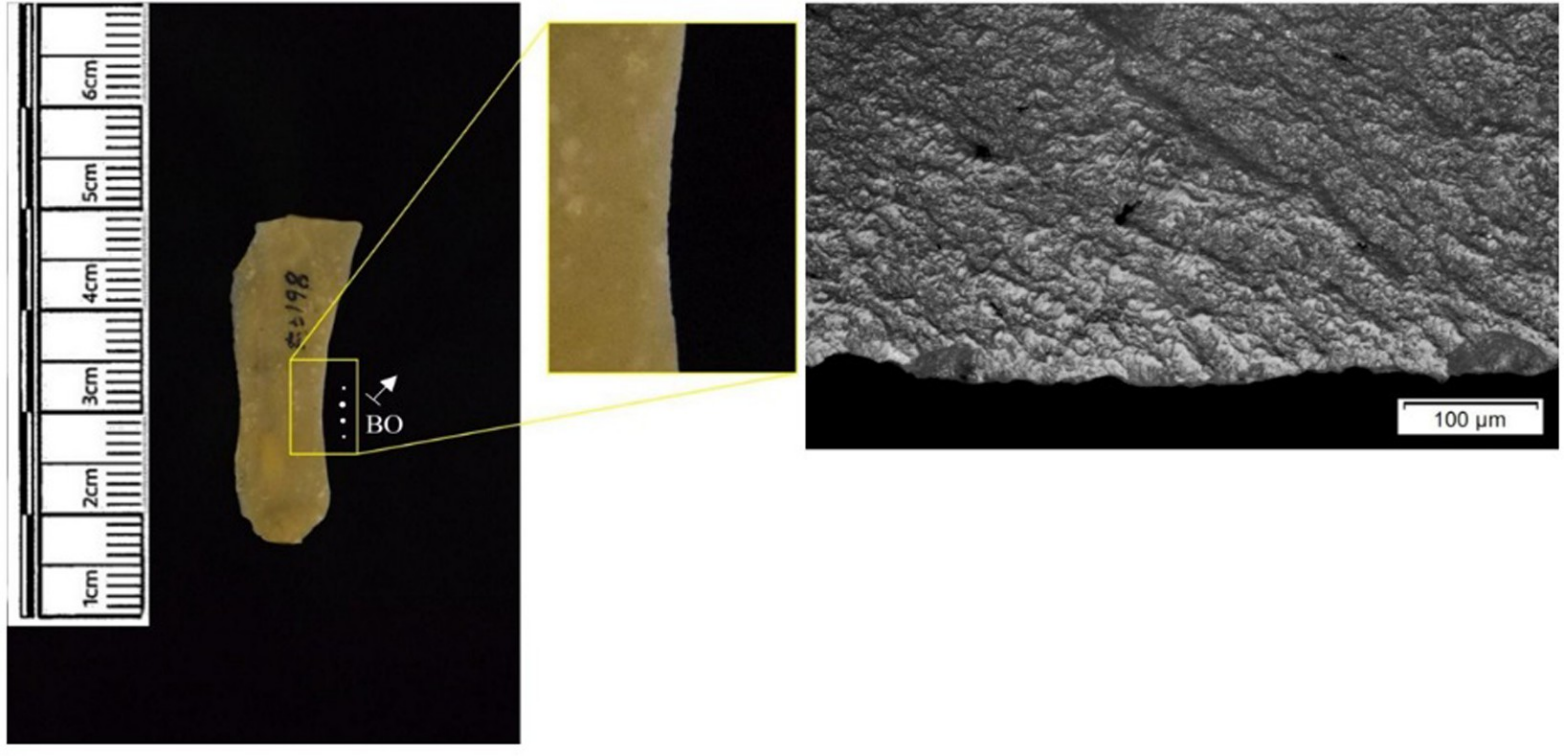
SC 86197, a curved bladelet interpreted as showing developed bone polish from scraping on ventral aspect, 200x magnification. No polish was observed on the dorsal aspect.

**Fig 21:**
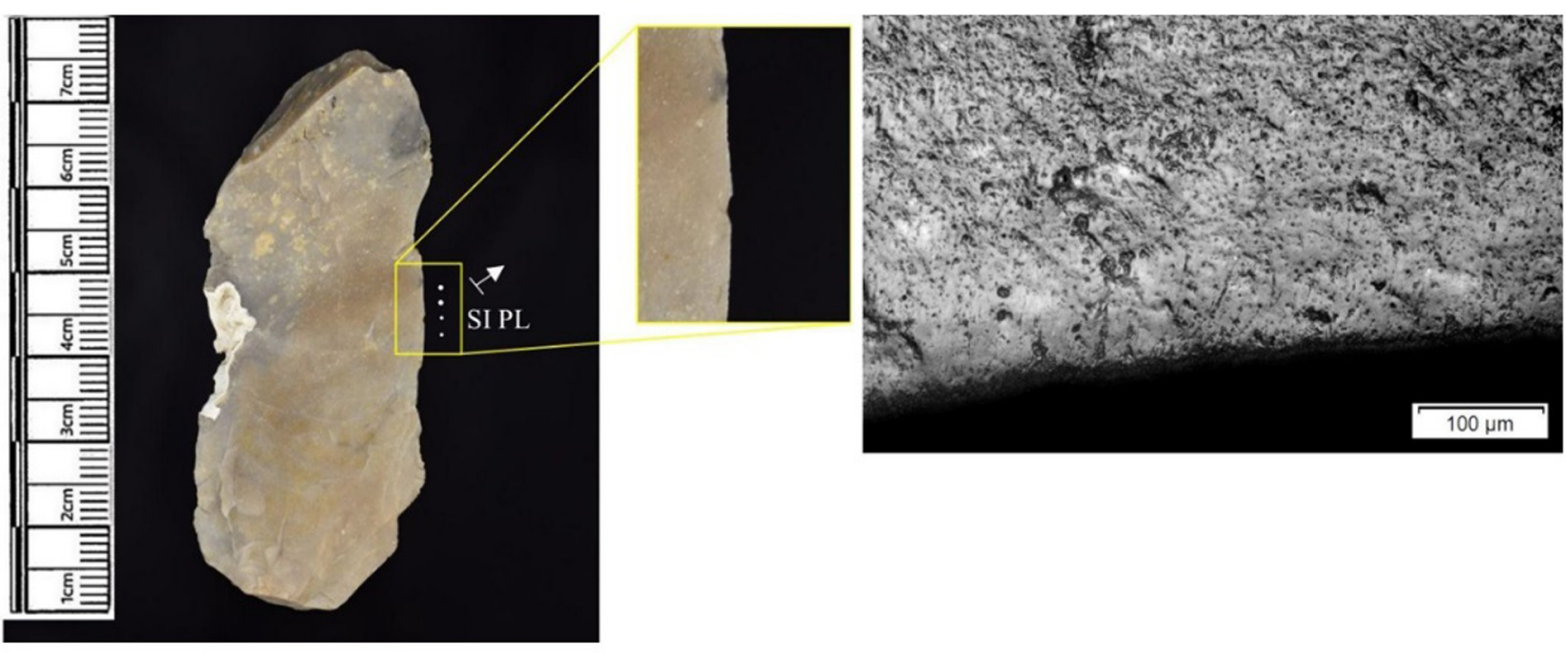
SC 90866, a blade interpreted as used to scrape siliceous plants, 200x magnification. The dorsal aspect did not show observable signs of polish.

**Fig 22:**
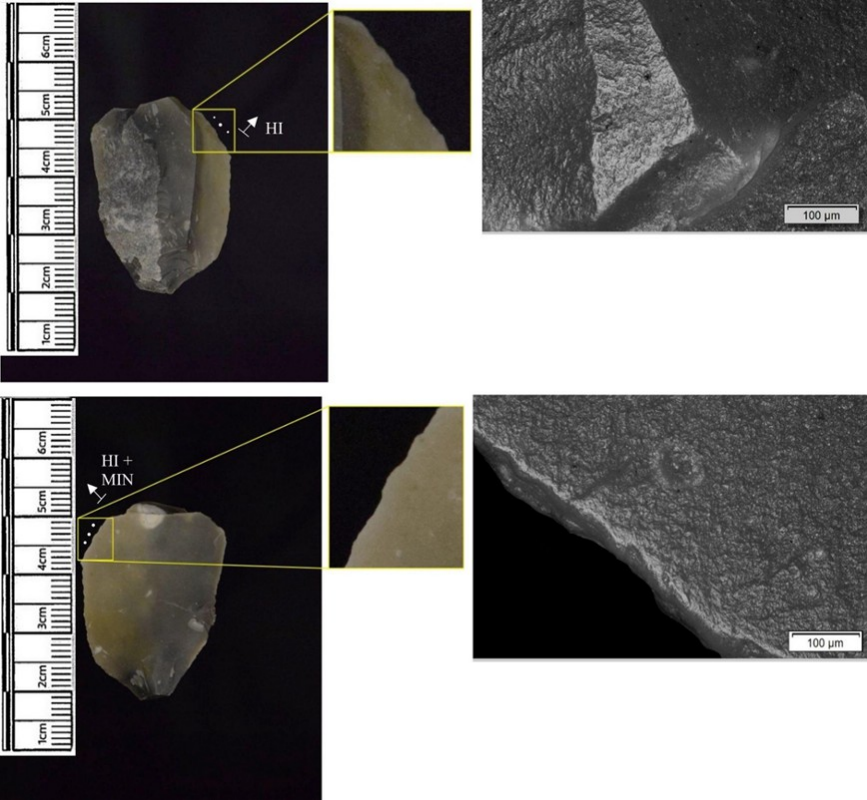
SC 84660, a scraper interpreted as used to scrape dry hide with an additive, likely mineral, 200x magnification. The polish appears more developed on the ventral aspect.

**Fig 23:**
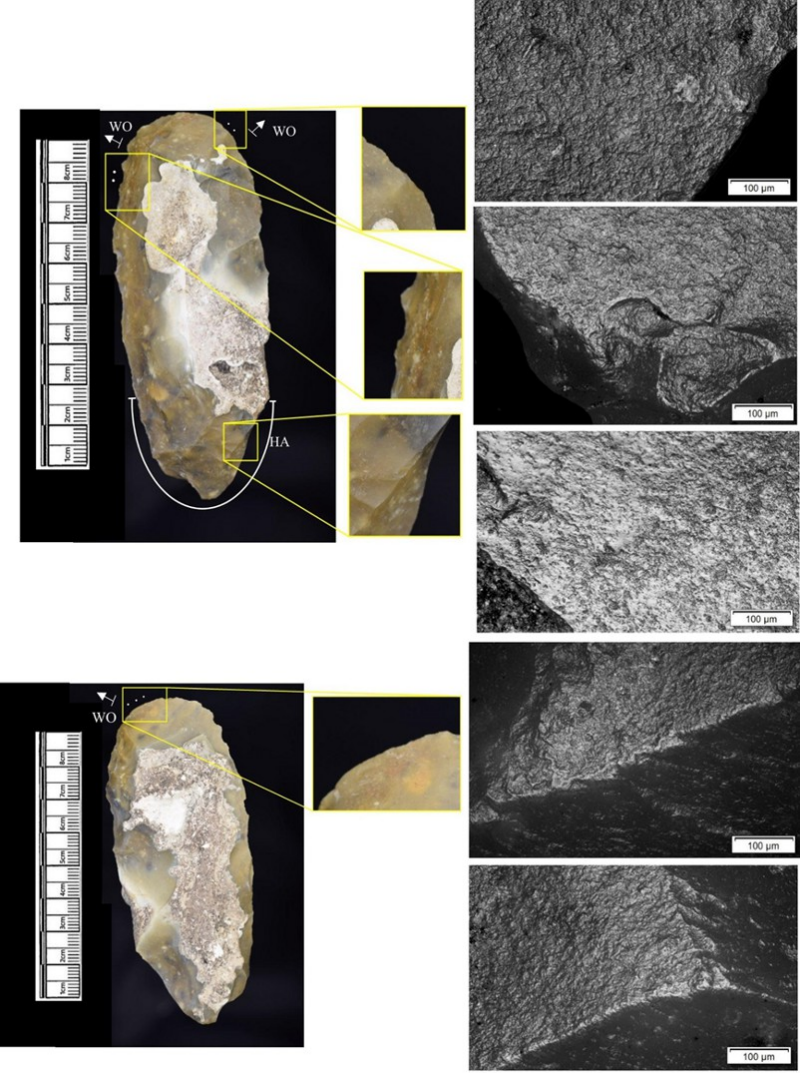
SC 86473, a tranchet axe interpreted as used to chop wood and hafted, possibly in a wooden haft, 200x magnification.

**Fig 24:**
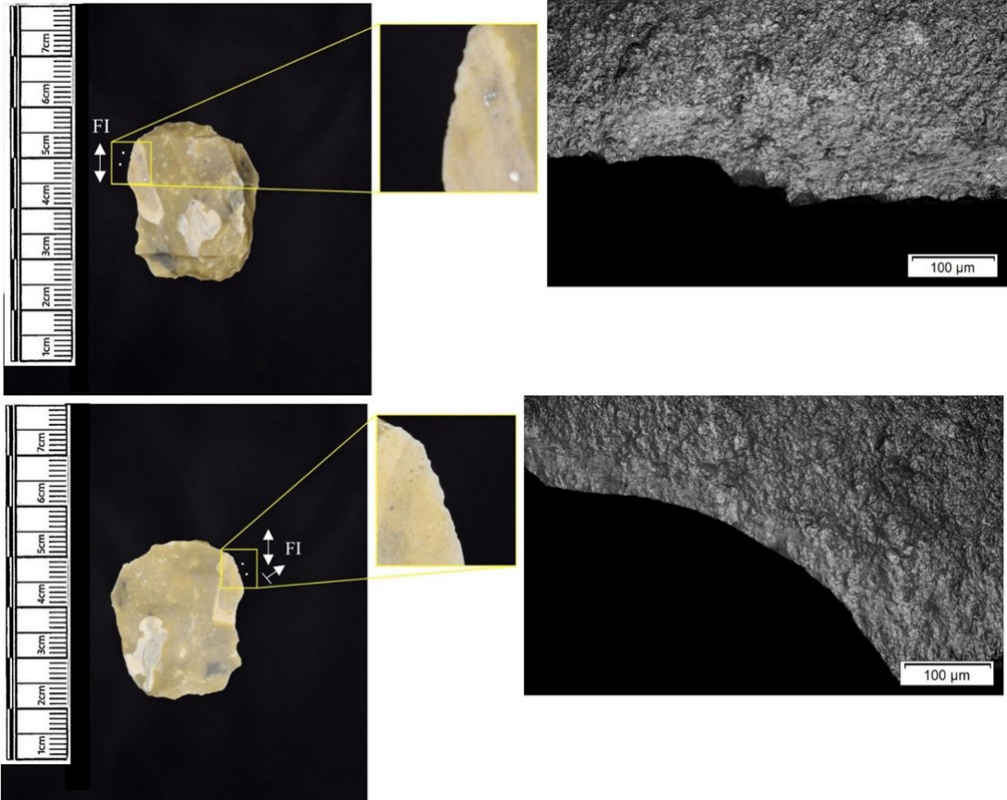
SC 91420, a scraper interpreted as used to possibly scrape and cut fish, 200x magnification. Striations can be observed within the polish, indicating contact with a harder material like scales [81].

In total, 146 flint artefacts were analysed from the eastern structure and its surrounds; 52 (35.6% of the total analysed sample) were located within the hollow. From those assessed, 52 (35.6%) were interpreted as not used. Rates of use and PDSM are unlikely to be representative of all lithic material, as 80 were selected during the second sampling phase. However overall, there were good levels of microwear preservation and low levels of PDSM. Wear traces were observed on 64.4% of pieces. The frequency of PDSM was generally low; 54 (37%) evidenced iron oxide staining or deposits, 39 (27%) flat dull smoothing, and 22 (15%) metallic striations. A range of materials were worked (Table 3). Bone was the most frequently worked material (n = 15 –Fig 20), followed by meat (n = 10), plant (n = 8 –Fig 21), hide (n = 8 –Fig 22), wood (n = 6 –Fig 23), use as a projectile (n = 5), antler (n = 1), fish (n = 1 –Fig 24).

**Table 3:** Summary of tool use in the eastern structure.

Primary contact material	Secondary contact material	Motion of use	Number
Antler		Transverse	1
Bone		Transverse; longitudinal	11
Bone	Meat	Transverse; longitudinal	3
Bone	Hide	Transverse	1
Fish		Transverse; longitudinal	1
Hide		Transverse; perforating	6
Hide	Soft mineral	Transverse	2
Meat		Longitudinal	4
Meat	Bone	Longitudinal	6
Projectile	Bone	Longitudinal (hafted)	3
Projectile	Meat	Longitudinal (hafted)	1
Projectile	Hide	Longitudinal (hafted)	1
Plant (inc. plant/soft wood)		Transverse; longitudinal	7
Plant	Meat	Transverse; longitudinal	1
Wood		Transverse; longitudinal	6

## Discussion

### Overview

There are two caveats to consider when interpreting this data. Firstly, any spatial patterns in tool use are only representative of the analysed artefacts, which are a sample of the overall assemblage. It is possible that further analysis of additional pieces would enable further insights into tool use and the spatial patterning of activities. Secondly, lithics may not have been used where they were excavated. An individual may have used a tool elsewhere and deposited it near to the structures, or flint could have been moved around site inadvertently or through post-depositional processes, meaning that tool use may not reflect activities undertaken near to or within the structures. However, this can be explored further through spatial patterning of tool use in relation to the possible boundaries of a structure. These limitations are present in any study that employs microwear and spatial analysis, particularly where the temporal sequence of habitation levels is not well preserved or defined. Figures presented in this discussion combine new microwear results alongside the previous interpretations of tool use by AL.

### Activity areas across the site

To interpret tool use within and around the structures, it is first necessary to summarise tool-using activity patterns from across the rest of the site. In doing so, the range of activities and spatial patterns can be more fully assessed as typical or not when compared to other areas that are not associated with structural features.

Overall, there are only a few instances where flint scatters indicate *in situ* and discrete activity areas from across the site. This is seen through previous typological assessments, refitting and microwear analysis [35]. These, as elsewhere in the Vale of Pickering, tend to be located in peat on the edge of the reedswamp where discrete tasks were undertaken away from the main living areas, but also buried relatively rapidly, meaning that the direct superimposition of activities, middening practices and scavenging of material characteristic of the dryland did not take place. Elsewhere on site, scatters are characterised by mixed tool types, containing little to no refitting sequences. Microwear results show that a range of contact materials were worked, and these microwear traces do not cluster spatially based on contact material.

An unusual, extremely dense cluster of 1024 pieces of tools and debris was found to the north of the central platform; a lake-edge platform made from large split timbers [35]. It was interpreted as an axe workshop based on the higher frequencies of axes, axe manufacturing debris, and resharpening flakes. Six axes in total were found, and only one displayed signs of use (wood working). A resharpening flake was also examined but it did not reveal any patterns of use [35]. Refitting rates were high in this scatter and suggest that this activity was localised to a specific area, with no obvious structural features to act as a physical container.

A crafting area to the south-east of the western structure features was identified from a flint scatter comprising 580 lithics. A higher number of awls were found, interspersed with utilised pieces, burins, scrapers and microliths. Microwear traces from 15 tools evidenced bone and antler grooving and scraping (7), as well as mineral (3), plant (3) and wood working (2) [35]. Despite evidencing a range of contact materials, motions of tool use were similar and suggest craft-related activity (i.e. perforating, engraving). Spatially, these pieces were intermixed and located in close proximity away from the structural features [82].

### Activity areas associated with the structures

#### Central structure

Spatially defined areas of specific tasks are difficult to identify due to limited identifiable microwear traces. These complexities are further exaggerated as the full extent of the structure is unclear [58]. Very few flints cluster together, so any related tool use could be interpreted as short episodes of activity, or just coincidence, rather than sustained structured behaviour. Microwear traces from working bone, hide, fish, meat and antler were inferred. Preservation of faunal remains in and around the central structure is generally poor, owing to high levels of burnt and calcined bone [66]. Therefore, activity areas associated with specific materials were not established for the central structure.

**Fig 25:**
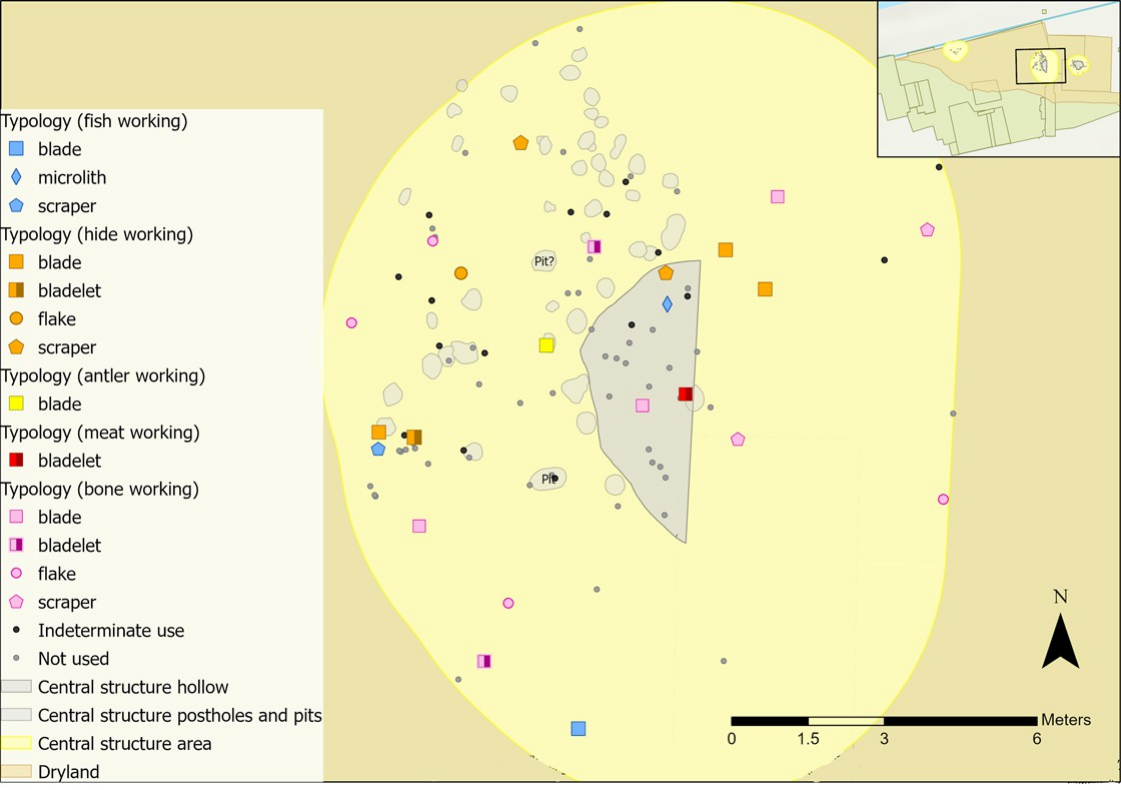
Animal-related tool use within the central structure sampling area with indeterminate and not used pieces plotted.

Bone working flints were largely dispersed with no clear spatial clustering or patterns in tool type. Five were located away from the hollow to the west and south-west, of which four were interpreted as used longitudinally on bone. This could indicate some homogeneity in the types of tasks undertaken. Two flints overlap the hollow of the structure (Fig 25), a blade used longitudinally on bone and meat, and a scraper with longitudinally orientated bone working traces. Meat and bone polish was also found on a bladelet in this area (red square on Fig 25). These tools might have been connected in butchery-related tasks, based on their use and spatial proximity; however, overall there is no clear spatial pattern in bone working tools in and around the central structure.

A group of three hide working tools associated with the hollow had both fresh hide and dry hide (with a possible mineral additive) microwear traces. Different stages of hide processing were observed and may not have been spatially defined. Alternatively, these tasks could have been undertaken at different times with the tools coincidentally deposited close together. Four tools used for dry hide scraping were located to the east of the hollow, of which two had soft mineral working traces. Polish from bone and meat working was also found in this area, along with a possible fish working scraper used to cut and scrape fish. Fish skin can be used similarly to hide; fish leather is as durable and effective as large animal hide so it may have been processed and utilised at Star Carr [83,84]. Microwear traces from working different states of fish skin (i.e. fresh, dry, tanned) are still not well understood, so it is unclear if the possible evidence of fish scraping was used on dry or treated skin. Tools used on hide and fish were dispersed across the central structure hollow and surrounding features.

**Fig 26:**
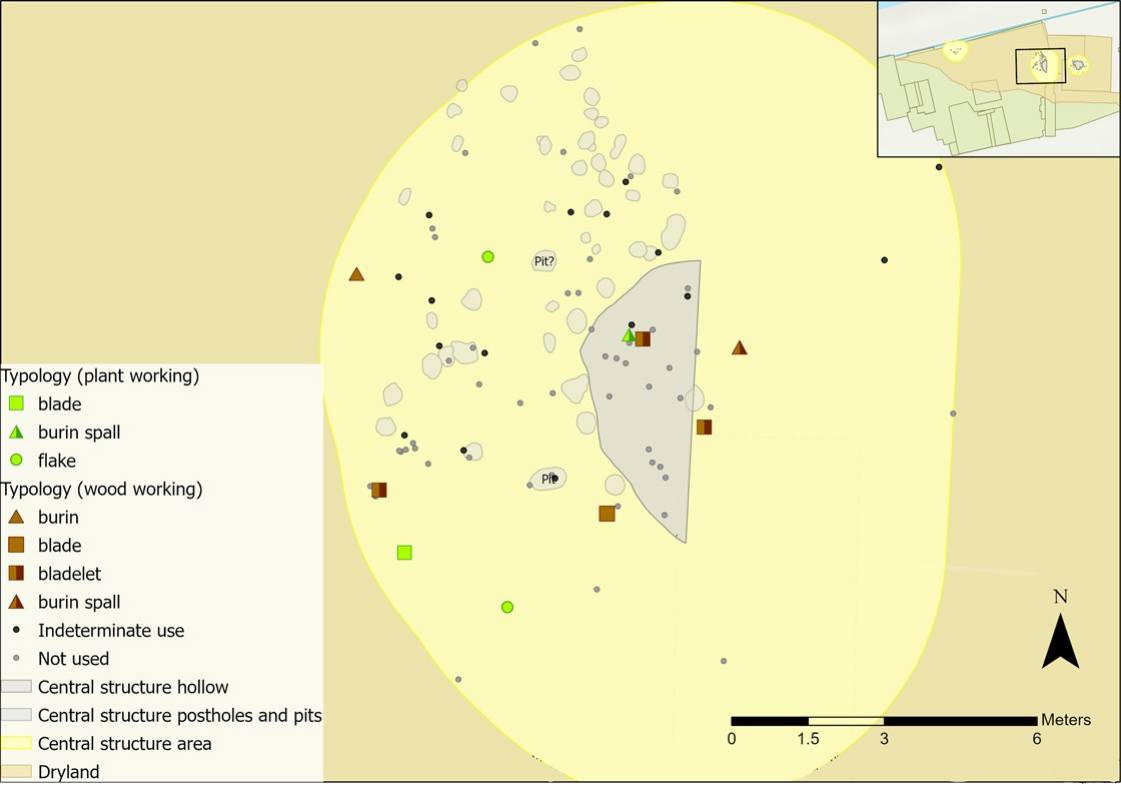
Vegetal-related tool use within the central structure sampling area with indeterminate and not used pieces plotted.

All woodworking tools had transversely orientated polish, with one bladelet also showing longitudinally orientated polish. These flints displayed wear traces that could indicate different stages of crafting wooden objects; planing, graving, and perforating. Of the wooden objects found at Star Carr, handles, dowels, digging sticks would have required planing and at least two objects had holes in, likely requiring perforation [85]. Microwear traces of plant or woodworking were spatially dispersed so activity zones were not established (Fig 26).

**Fig 27:**
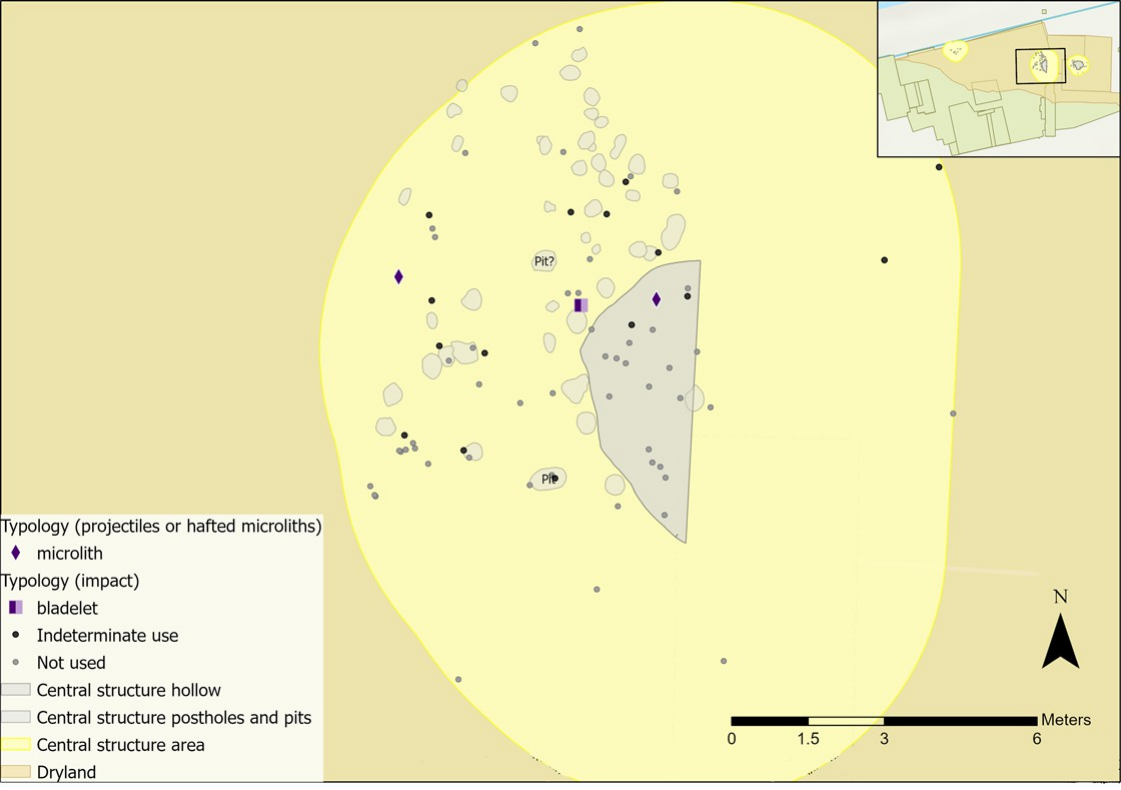
Flints interpreted as projectiles or hafted tools found within the central structure sampling area with indeterminate and not used pieces plotted.

The spatial distribution of hafted tools and those with microscopic linear impact traces (MLITs) is more convincingly interpreted as reflecting de-hafting or de-commissioning practices, rather than where they were last used [86]. These tools might be expected to cluster spatially if they were dehafted or retooled in a similar location, as observed at other Mesolithic sites [87,88]. For example, dehafting can occur around a hearth, as heat is used to soften adhesives and loosen flint inserts [57,86,87]. Both microliths found in the central structure suggest use as hafted composite tools, either projectiles or as part of a tool like a knife. The microlith located in the hollow may have been de-hafted in the area after use. There does not appear to be related activity occurring here as both tools were dispersed (Fig 27). The bladelet analysed by AL had signs of impact, indicating use as a projectile, so it is possible that some composite tools may have been dehafted in this area.

**Fig 28:**
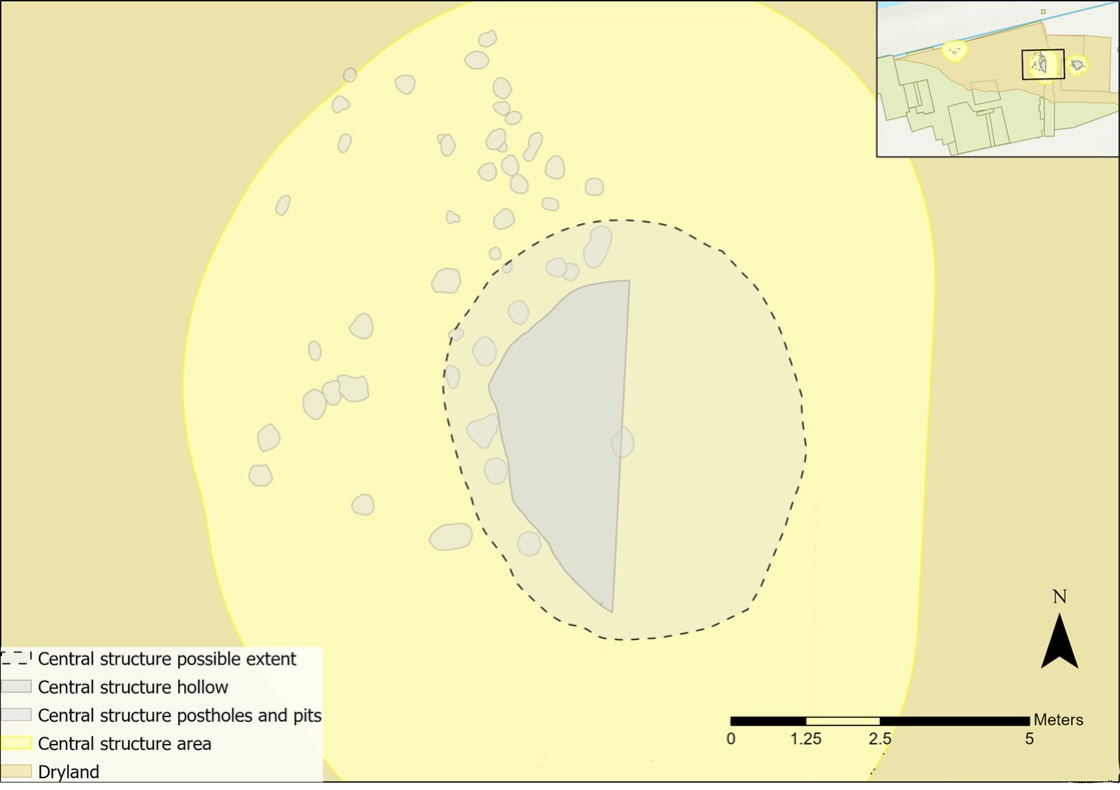
Proposed extent for the central structure.

A proposed boundary for the central structure is based on the shape of the features (Fig 28). Microwear results did not provide sufficient data to suggest that any activity was confined to a particular area. Other postholes may not have been identified during excavations, which makes the area complex to interpret. Taking these complexities along with the likely clearance activity associated with it, it is possible that the activities inferred may have post-dated the structure [35]. Using the features, a mirror-image of the hollow and associated postholes was used to establish a minimum boundary (Fig 25).

A cluster of three hide working tools and a scraper with possible fish polish were located near to the western arc of features, and one was found within a possible posthole [58]. A rack or frame may have been constructed to aid the scraping of hides, or a drying rack to smoke skins for tanning. Microwear traces also suggest that tools in this area were used in tasks relating to animal skin processing.

The central structure is considered to have been a substantial post-built structure. Only a portion of the hollow was excavated, but the features indicate that it would have provided some level of shelter and could have withstood the elements. Evidence of activity is generally sparse, with limited flints, refits, faunal remains and indications of clearance from geochemical analysis. A sparsity of flints within the structure creates difficulties in interpreting tool-using activity. This may have been a structure kept clear of flint, with those pieces analysed highlighting only a portion of the overall activity. Alternatively, activity may have taken place after the structure was abandoned, with microwear results mirroring the interspersed spatial patterning of tool use observed elsewhere in the dryland. Some Mesolithic structures with few associated flints have been interpreted as dwellings owing to the sparsity of finds [e.g. 89]. Conversely, Grøn [47] has argued that even if clearance activity occurred, lithics would be expected from the last use of the structure prior to its abandonment.

From all the activities inferred within the central structure area, wood and hide working tools were most commonly associated with the hollow and postholes. Four pieces with wood working traces were used to groove or engrave wood or plane a small piece of wood; actions indicative of small-scale activities. One of the hide working tools was used to scrape dry hide with a possible mineral additive, which could indicate tanning or further hide preparation. If the flint is associated with the structure, it is possible that it was kept clean and used only to process certain materials, possibly relating to craft working, similar to a workshop [38,69].

#### Western structure

**Fig 29:**
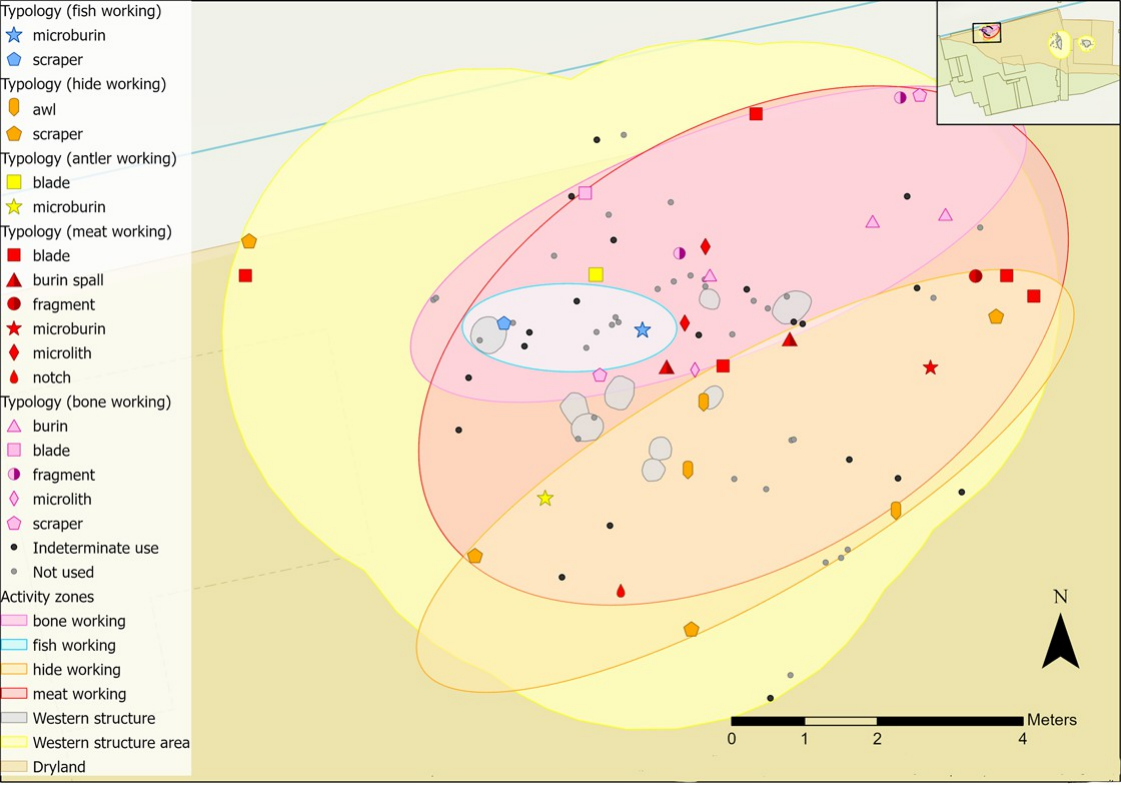
Animal-related tool use within the western structure sampling area with indeterminate and not used pieces plotted.

The flints related to bone working fall within a relatively small area within the north-eastern part of the sampling area, and to the north of the potential postholes (Fig 29). Activity in the western structure presents a combination of processing of bone for subsequent uses or craft work, as well as butchery. Most tools with meat working traces were cutting tools, indicative of butchery and these overlap with bone and hide working tools.

Near to the features, a fragment and scraper were used to scrape bone. This may reflect cleaning of excess fleshy material, suggesting that bones may have been prepared here prior to further processing such as making bone implements. To the north-east of the features four bone working tools (two burins, a scraper and a fragment) were used to scrape and to groove/engrave. In contrast, the hide working tools are mostly located south of the butchery and bone working tools. This evidence comprises five scrapers which fall outside the potential postholes and three awls used to perforate hide.

Finally, two flints exhibited signs of fish working and were located within the potential post-holes (Fig 29). The scraper was used to cut and scrape fish, actions that could relate to processing fish skin; however, this was not found in association with the hide working tools.

**Fig 30:**
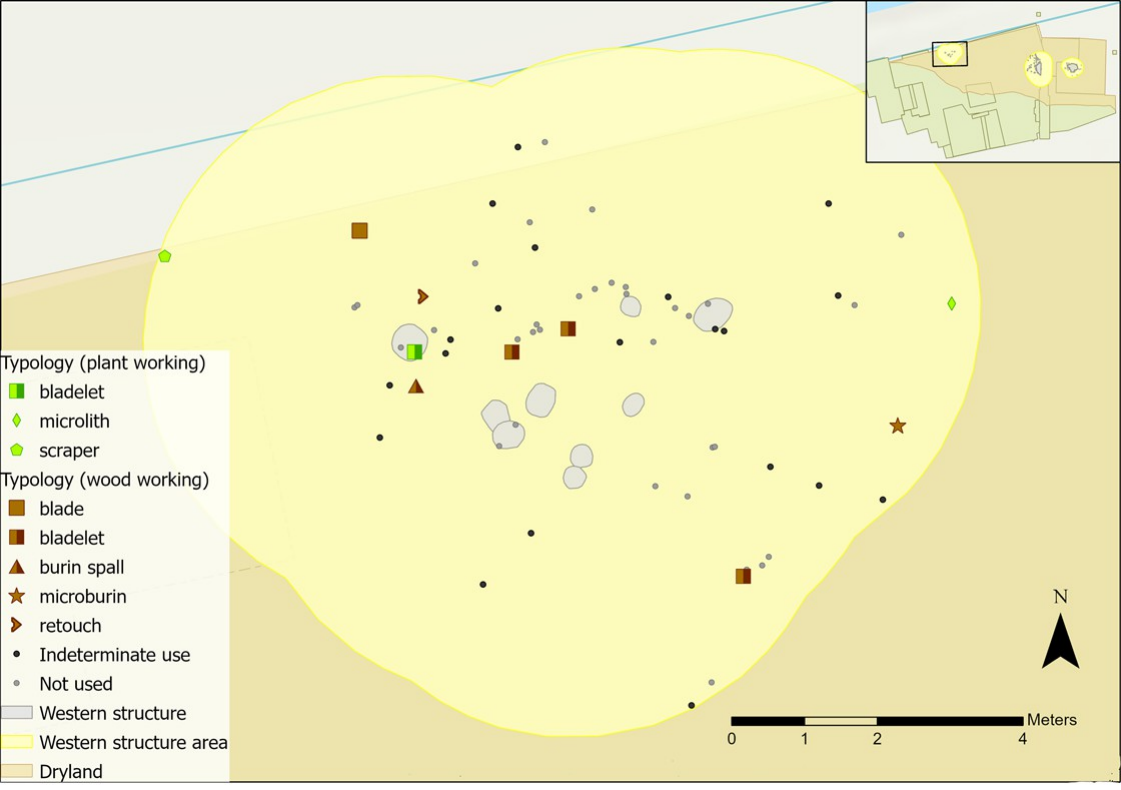
Vegetal-related tool use within the western structure sampling area with indeterminate and not used pieces plotted.

Activity zones were not established for plant or wood working as flints were too scattered (Fig 30). Four of the wood working tools found closest to the features have different microwear traces, with both hard and soft wood displaying longitudinal and transverse directionality. These actions are indicative of cutting, scraping/graving and debarking, and are more likely to relate to crafting objects because of the size of the tools. A bladelet found within the features was used to cut and scrape siliceous plants, which could also be interpreted as craft-related activity (the scraper was previously analysed by AL).

**Fig 31:**
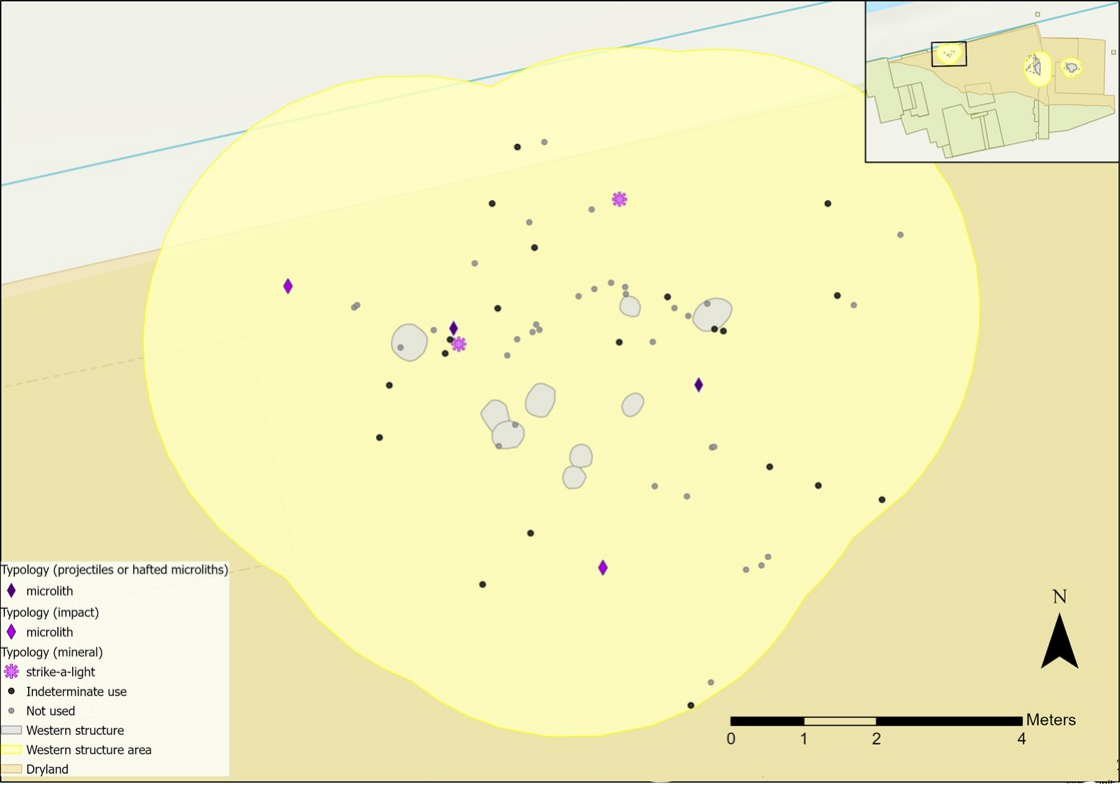
Flints interpreted as projectiles or hafted tools and those with mineral microwear polish found within the western structure sampling area with indeterminate and not used pieces plotted.

Four microliths were interpreted as projectiles and possible MLITs were observed; two were previously analysed by AL (Fig 31). Both pieces examined as part of this research were interpreted as having contact with bone and meat, as expected if used for hunting. Some MLITs were found on the dorsal aspect towards the distal tip of one piece, suggesting use as a projectile tip (Fig 17). One piece analysed by AL was used to cut plants once it had been dehafted, which may suggest ad-hoc *in-situ* reuse of flints in this area.

Two strike-a-lights were recovered from the sampling area (Fig 31), though there was no evidence of an *in-situ* hearth [35]. Neither had signs of burning, and faunal remains also showed no exposure to heat.

**Fig 32:**
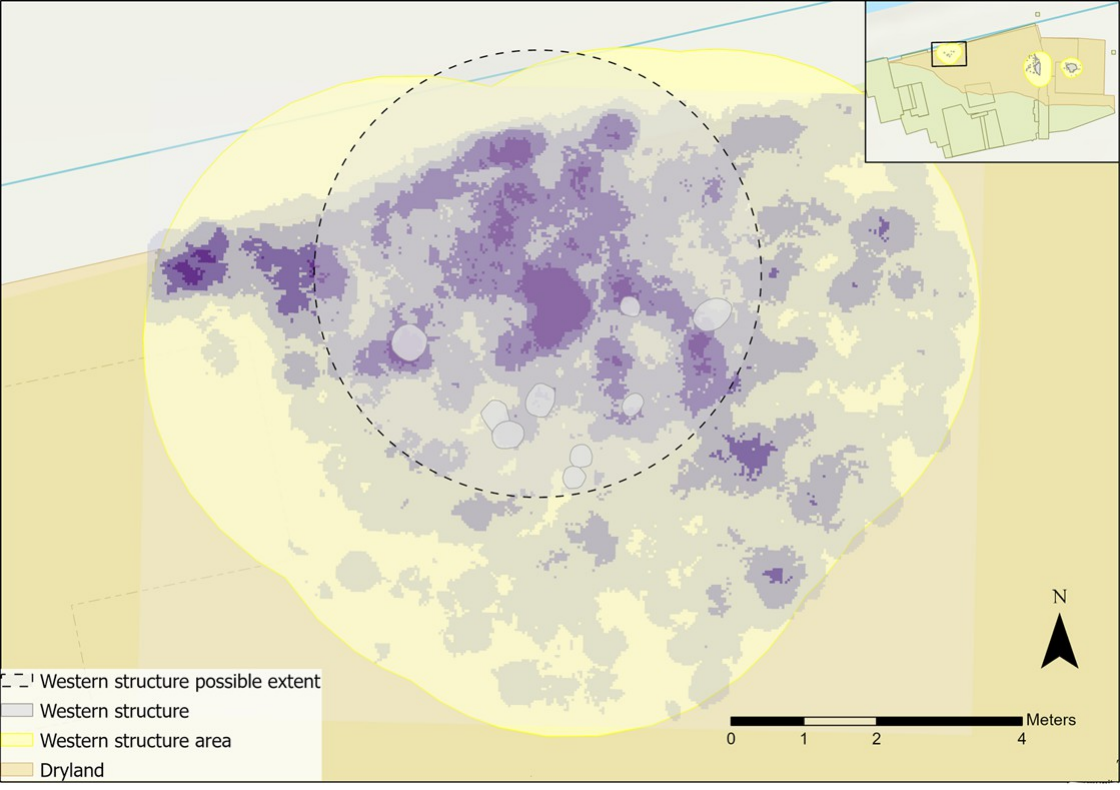
Flint density and western structure features with possible extent of the structure indicated with a dashed outline.

The features alone do not aid interpretations of the structure’s boundary as only two post holes were identified with certainty [58]. Post holes may indicate central weight-bearing posts of a structure that was rebuilt or remodelled during its use, be the southern limits of a structure that extended beyond the trench, or perhaps relate to a frame for a drying/smoking rack or windbreak. A higher flint density was located to the north of the features, with significantly lower densities to the south and west (Fig 4–section 1.2.3). Higher densities extended up to the area that is now the Hertford Cut, suggesting unexcavated activity areas, and perhaps features, are located there. A tentative boundary for the western structure can be suggested from plotting the flint density within this area (Fig 32). It is also possible that the densities of flint occurred from midden activity; the secondary deposition of material in one area could also create a similar density pattern even without the constraints of a structure [90].

Limited and uncertain identification of features excavated in the western structure area make interpretations problematic. There remain ambiguities, notably the nature of the structure (i.e. whether it was a rack or more substantial structure) and when the material was deposited (i.e. when a structure was present or not). It is probable, from the current distributions of flints and faunal remains, that the structure extended north beyond the field boundary into the bank of the canalised River Hertford. Therefore, the materials and features excavated are potentially only a part of a much bigger activity area, skewing interpretations.

It is most likely that this area was later used as a midden–its last use before abandonment–with clearance material from other areas deposited here [35]. The mixed treatment of flints and animal bones could indicate the presence of a midden, where tools from different areas (some from cleared hearths) were deposited. Based on the densities of flints and faunal remains extending to the north, there may have been a more substantial structure present, perhaps a dwelling, that was subsequently deserted and repurposed. Microwear results indicate that some tasks like bone and hide working were spatially distinct; however, craft-related activity was interpreted in both of these groups. Craft work on different materials may have been undertaken in specific areas in and around the western structure, with hide working to the south of the features and bone working to the north. It is possible that these activities may indicate *in-situ* tool use, either associated with a structure or a later midden and undertaken in different areas.

#### Eastern structure

**Fig 33:**
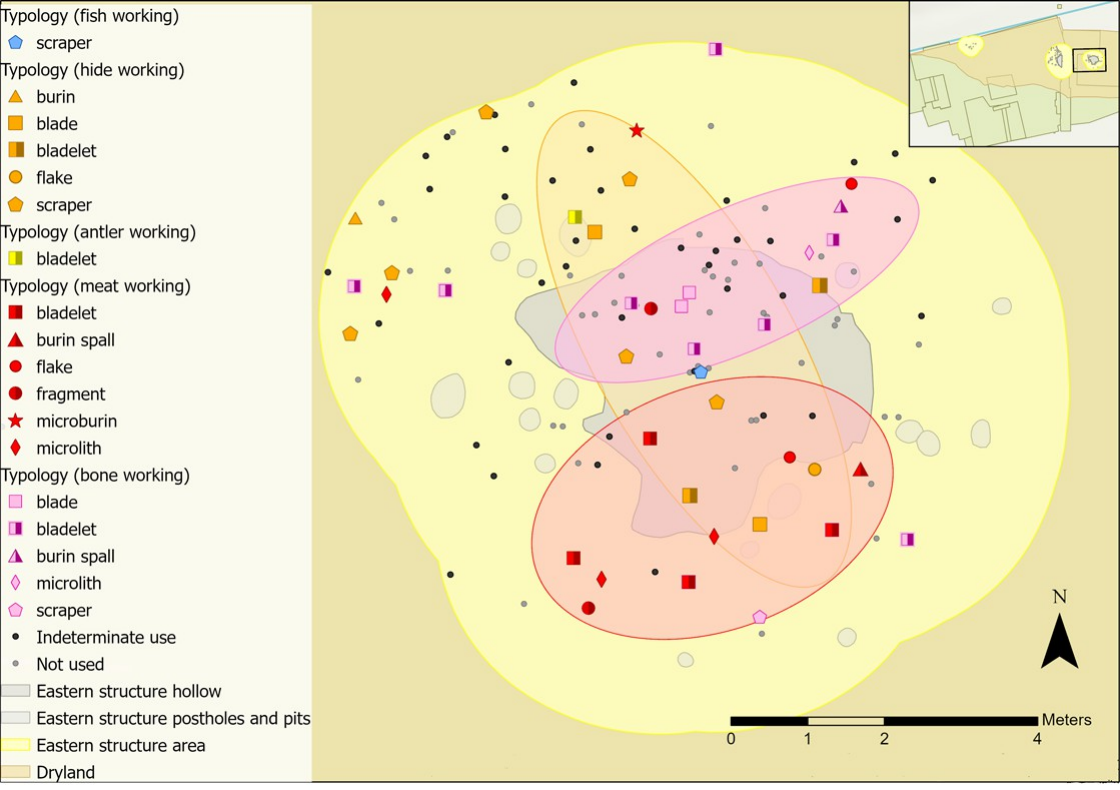
Animal-related tool use within the eastern structure sampling area with indeterminate and not used pieces plotted.

Animal-related polishes in the eastern structure comprise bone, meat, antler, hide and fish. Zones of activity were not established for antler and fish working as too few tools with these microwear traces were found. A distinct zone of meat working was established in the southern half of the study area (Fig 33). Tools used to work bone were mostly located in the north half of the hollow and beyond and meat working was mostly located in the southern half of the hollow and beyond. Meat and bone are often interlinked in tool use (for example, in butchery, as was observed in the western structure), so it is interesting that bone and meat working zones are spatially defined (Fig 33).

Hide working on the other hand mostly occurs in the hollow and to the northeast but overlaps with the bone and meat working zones. Of the eight hide working tools in this activity area, four located to the south were used to scrape fresh hide. Two scrapers in the northern area were used to scrape dry hide, a bladelet scraped possibly fresh hide and a blade pierced dry hide and soft mineral. Largely these activities likely reflect later stages of hide processing, where dry hide is worked, sometimes with mineral additives to produce containers, clothing and coverings. Therefore, the area to the south of the structure could have been used for processing fresh animal products, like meat and animal skins and the northern half used for mainly bone working tasks and dry hide work.

One flint was found with evidence of fish processing, like those from scraping fish scales and skin, as the scraper had striations within the polish, indicating contact with a harder material like scales [81,91,92]. This suggests that fresh fish was processed, with scales likely removed prior to drying, smoking or cooking and is reinforced by 10 fish bones found in the structure, nine of which were burnt, suggesting cooking [66,84].

Possible antler polish was observed in the northern half of the hide zone on a bladelet. Barbed antler points, antler mattocks, and frontlets would have all required processing, likely with flint tools [93]. Rather than use on larger, complete antlers in the early stages of splitting, owing to its size, the bladelet could have been used in the final phases of barbed point production. The barbs and point would need defining by planing excess antler away [94,95]. A fragment of antler was found in the structure, reinforcing that antler working was an *in-situ* activity [66].

**Fig 34:**
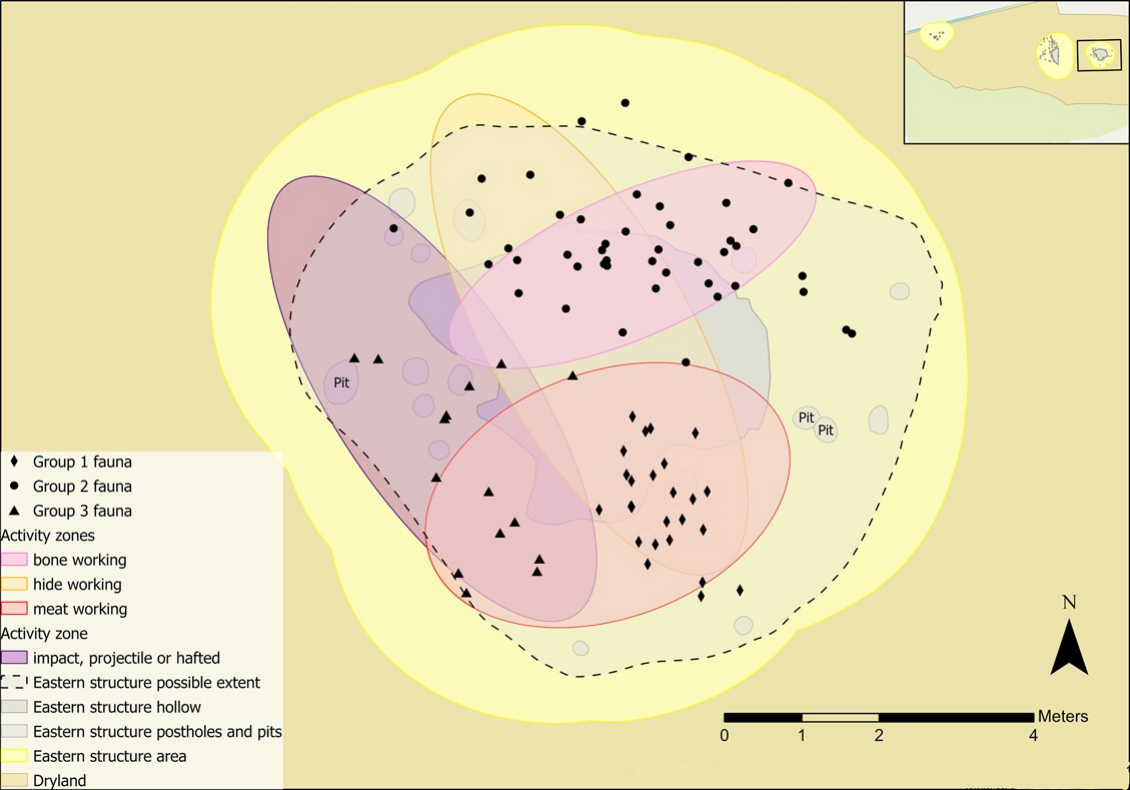
Distribution of faunal remains within the eastern structure sampling area.

Faunal remains found in close association with the eastern structure mirror several of the zonal areas; two concentrations (Groups 1 and 2) correlate with meat and bone tool-using activity (Fig 34). These concentrations are similar in character, with large quantities of highly fragmented specimens, mostly identified as cervids, with some evidence of charring or heat [66]. The only notable difference is that the majority of one bone assemblage (Group 1, located in the eastern part of the meat working zone in Fig 34) did not display human modification. This contrasts with Group 2, which had signs of spiral fractures, percussion breaks and/or longitudinal splitting [66]. Breaks and longitudinal splitting of bones can be an initial step for crafting bone objects.

**Fig 35:**
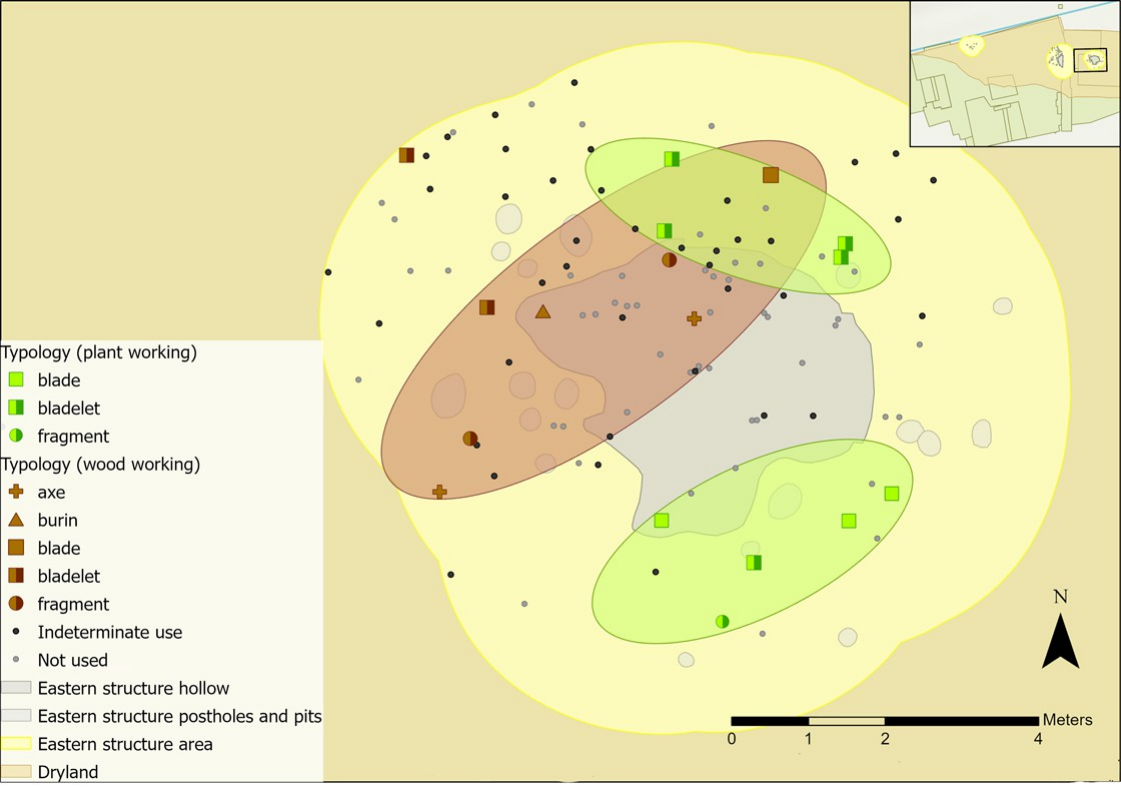
Spatial distribution of tools used on vegetal-related contact materials and associated activity zones in the eastern structure sampling area.

Microwear traces from plant working were found to the north and south of the hollow, grouped into two zones (Fig 35). Of those in the southern group, three were used to process siliceous plants (e.g. reeds, sedges), whereas those in the northern half were all used on non-siliceous plants (e.g. nettles). Plant type is not likely to correlate to tool type, as bladelets were found in both groups with different types of plant working traces. Patterning might instead suggest that plant working was organised based on the types of plants processed. This might reflect single episodes of activity rather than sustained patterns of behaviour. Plant residue from silica-rich species can stick to the flint’s edge, creating a blunting effect that cannot be wiped off easily. This means scraping siliceous plants can result in high quantities of exhausted or blunted pieces [96]. As an example, the processing of reeds over a short duration may result in using three tools within one small area.

Wood working traces were found in the north-western areas of the hollow and beyond (Fig 35), with different tool types and directionalities represented. Cutting, scraping, planing and engraving actions were observed, indicating a range of tasks were undertaken such as crafting wooden objects, debarking wooden stakes, or processing firewood. In the wetland areas, numerous wooden objects were found, including at least three hafts or handles, dowels, stakes, digging sticks, a willow and roundwood withy and a bow [85].

**Fig 36:**
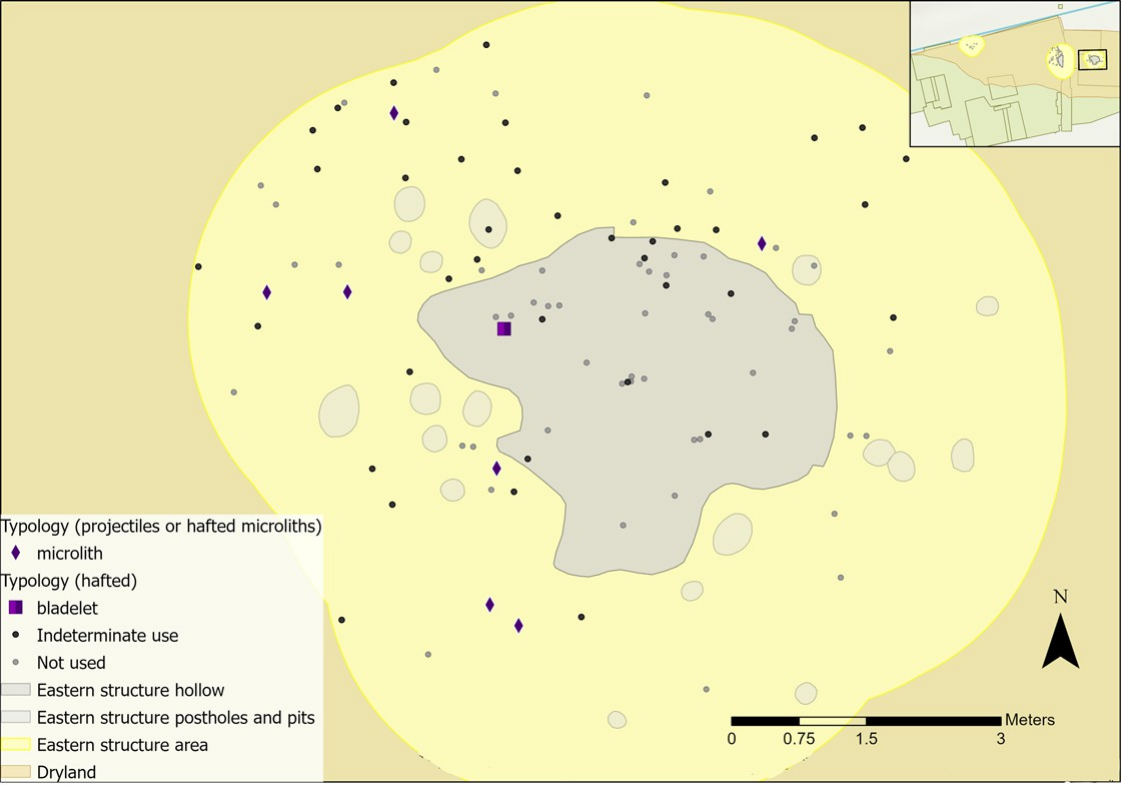
Flints interpreted as projectiles or hafted tools found within the eastern structure sampling area with indeterminate and not used pieces plotted.

All pieces with hafting or projectile microwear traces had longitudinally orientated polish and so were interpreted as hafted to the side of a projectile or knife handle. Most projectiles/hafted microliths were located outside of the hollow (Fig 36). They are largely dispersed with no clear localised area of dehafting and/or use. Two microliths located to the south indicated contact with bone and meat and hide, which could be consistent with them being hafted into the same projectile.

**Fig 37:**
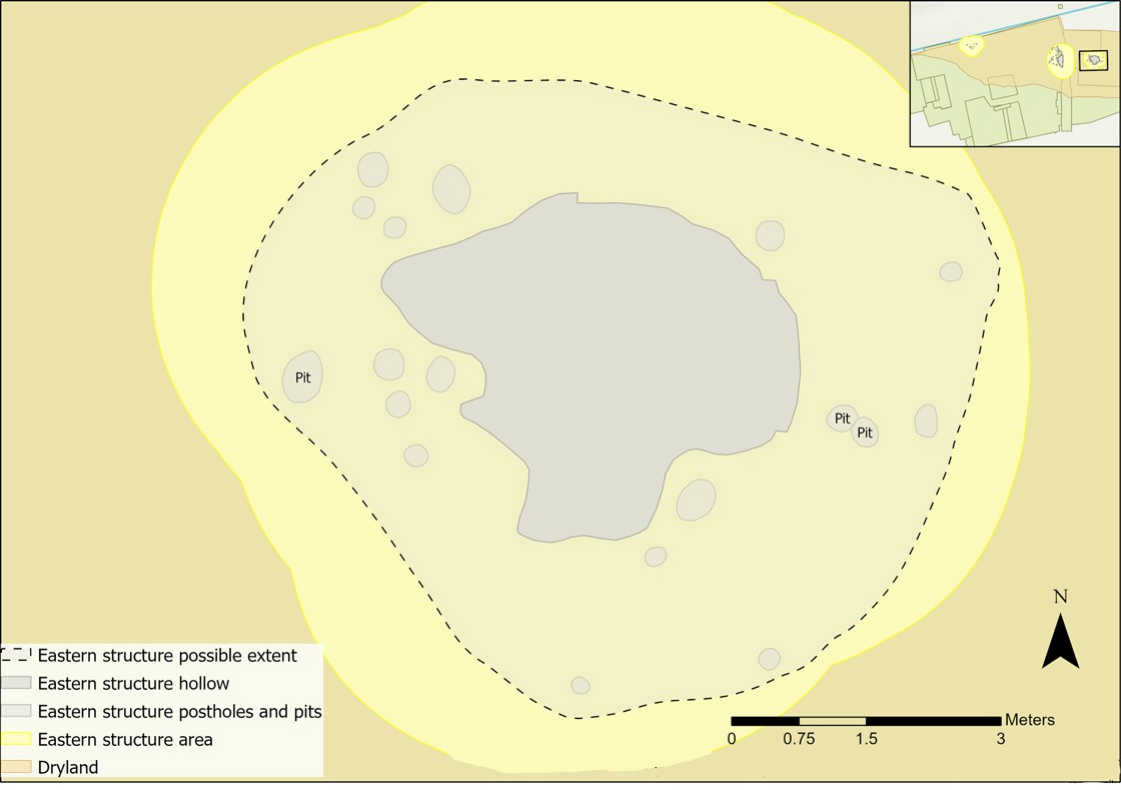
Proposed extent for the eastern structure.

Several postholes surrounding the hollow are likely to have held the main weight-bearing posts of the structure (Fig 37). Two clusters of four postholes to the west of the hollow could be interpreted as indicating structural maintenance. Posts may have been repositioned or changed as they deteriorated, similar to interpretations of Howick, Northumberland [97]. Alternatively, the clusters could indicate additional structural post supports for the main frame [58]. A boundary of the structure, which includes these features, was established. It is possible that this extended further out as, owing to the ephemeral nature of the postholes, additional outer postholes may have been present to the north and west of the hollow.

There is clear spatial patterning in the eastern structure. The working of bone and meat appear to fall into two zones to the north and south of the structure respectively. Some of this material may have accumulated over time, as well as *in situ* episodes of later phases of activity, yet these zones are notably distinct [35]. It is possible that the analysed pieces only reflect the last episodes of activity in the structure, thus creating distinct working areas, rather than a messy palimpsest from different depositions.

Spatial patterns in plant processing might reflect the differential treatment of plants compared to animal-derived materials, with perhaps less constraint on the location of plant working activity within a structure. Alternatively, these tasks may have been more ad-hoc and therefore less spatially structured compared to the working of other materials. It is important to note that interpreting the presence of plant working activity from flint tools alone is problematic. A lot of plant processing can be carried out without any tools, or with bone tools, leaving no wear traces on flints [98–101]. Unique spatial patterns in plant working using flint tools have been observed at other Mesolithic sites, such as Rosnay in France. There, tools with plant polish were more dispersed compared to those with butchery, hide and mineral working traces, which led to an interpretation of ad-hoc plant working on a specialised skin-processing site [102].

Comparisons to activity across Star Carr further highlight that the spatial patterning in tool use within the eastern structure is unique. This pattern is replicated by clustering in formal tools, with burins and burin spalls for example concentrated in the northern part of the structure [35]. This could suggest that inside space was organised in a specific way, with more clearly defined areas for working particular materials or that in contrast to the other two structures, this location was rarely used after the structure was abandoned. In the eastern structure, it is possible that tasks may have been organised due to practicalities. Spatially differentiating crafting, such as processing bone and possibly projectile hafts, and food processing might be expected as they are distinct tasks that produce different end products. Food processing and fresh hide working could also be considered messy activities, involving fresh meat, skins, bone and possibly blood. Delineated areas of activity relating to animal carcass processing separated from bone/antler work have also been identified at other Mesolithic settlements, though rarely within a single structure [34,38,103].

## Conclusions

Interpretations of tool use at Mesolithic sites often have a technological focus or tend to assign the whole settlement as a certain type (base camp, specialised camp, hunting camp) without examining and understanding tool use patterns associated with structures. This research presents a new integrated approach that explores tool-using activity areas as a means to more fully understand the social dimensions of Mesolithic sites. Key contributions of this paper can be summarised as follows:

1. Intra-site variability in Mesolithic structures should be anticipated.
2. Even in areas of high flint density, spatial patterns in tool use can be identified.
3. A ground-up approach (including individual tool use and spatial analysis) to activity in structures provides direct insight into the organisation of space and contributes to our understanding of cultural practices.

Dryland structures at Star Carr do not show similar patterns in use. In fact, it is difficult to discern many similarities apart from the range of contact materials worked, though even then, the western structure evidenced strike-a-light use, which was not seen elsewhere from pieces analysed. Inter-site variability in Mesolithic structures is to be expected; however, the level of intra-site variability identified at Star Carr is rarely observed at other sites. Excavated features and assessments of tool types showed some similarities between the structures but tool use presented a different picture. For example, strike-a-lights were identified in the western structure and a range of plants (siliceous and non-siliceous) were worked with tools found in the eastern structure. This contributes significantly to our understanding of Mesolithic structures and our expectations regarding how they were used. Consequently, we argue that variability in the ways structures were used, maintained (through clearance and depositing in a midden) and reused even within one site, should be anticipated. There remain, however, notable complexities in interpreting the ephemeral features and variable depositional practices associated with the structures at Star Carr. Despite this, through the integration of microwear and spatial analysis, it was possible to establish a likely extent of the eastern structure based on the features and activity areas. Results from the eastern structure demonstrate that it is possible to identify spatial organisation even where there are high densities of worked flint. Remarkably, these patterns in use are visible despite the palimpsest of activities present at Star Carr.

Few interpretations of Mesolithic dwellings present alternatives to Grøn’s approach to explain why activities may have been spatially structured [45,49]. Such spaces may have represented a family unit with fixed places for individuals, as Grøn suggests [47,48]; however, it cannot be considered the only possible narrative for the spatial organisation of Mesolithic dwellings. Tasks may have been undertaken in specific areas due to practicalities of the activities themselves. For example, butchery being a potentially messier activity compared to crafting with dry hides or producing bone tools. As patterns in activity are clearly distinct in the eastern structure, it is possible that the individuals who used the structure understood where each respective task should be undertaken. Behaviours inside the dwelling may have therefore adhered to accepted cultural practices, either specific to that dwelling or more general customs of the group. An alternative perspective on the organisation observed may therefore be through social norms, as even practicalities can be informed by notions of what is appropriate within a group (i.e. which materials can be processed in the same place). This presents a new avenue for discussing Mesolithic dwellings as more than just proxies for social units and for exploring intentionality in the organisation of space [104].

Applying the methodology presented here to other Mesolithic structures will contribute to a better understanding of how tasks were organised (or not) at different sites, within different contexts. Connections between Star Carr and settlements across the wider landscape of Britain have already been highlighted [105]. Inter-site comparisons where microwear studies have been applied would help elucidate whether this relationship also translates into the use of structures. In this way, microwear assessments can provide more nuanced data on the variability of Mesolithic structures or confirm/refute similarities to the observations made for Star Carr. From this, wider networks of communities and cultural practices relating to the organisation of space and use of structures could be explored at a larger scale.

## Supporting information

S1 Table Complete microwear data from all analysed pieces. (XLSX)
